# Cloacal microbiome variation in wild and captive Eastern Indigo Snakes (*Drymarchon couperi*) with and without *Cryptosporidium serpentis* infection

**DOI:** 10.1371/journal.pone.0350824

**Published:** 2026-07-09

**Authors:** Christopher Roger Brown, Mark Nikolaus Yacoub, James E. Bogan, Matthew D. Buehler, Michelle Lea Hoffman, Janina Alessandra Krumbeck, Zachary J. Loughman

**Affiliations:** 1 Department of Comparative Medicine, Tulane University, New Orleans, Louisiana, United States of America; 2 MiDOG Animal Diagnostics LLC, Tustin, California, United States of America; 3 Central Florida Zoo and Botanical Gardens’ Orianne Center for Indigo Conservation, Eustis, Florida, United States of America; 4 Department of Biological Sciences and Auburn University Museum of Natural History, Auburn University, Auburn, Alabama, United States of America; 5 Department of Biological Sciences, West Liberty University, West Liberty, West Virginia, United States of America; HUN-REN Centre for Ecological Research, HUNGARY

## Abstract

The Eastern Indigo Snake (EIS; *Drymarchon couperi*), a federally threatened species native to the southeastern United States, serves as a valuable model for examining the effects of captivity and infection on gastrointestinal microbial composition in reptiles. As an alternative to direct gut sampling, we examined the cloacal microbiomes of EISs to evaluate changes in microbial community structure across our study groups. This study assessed the cloacal microbiome of wild and captive EISs using shotgun metagenomic sequencing. Samples were divided into three groups for comparative microbiome analysis: captive snakes positive for *Cryptosporidium serpentis* (*C. serpentis*), captive snakes negative for *C. serpentis*, and wild snakes. Alpha (Shannon index, paired Wilcoxon test) and beta diversity (Bray-Curtis dissimilarity, PERMANOVA, CAP) metrics were used to assess microbial diversity and community composition across groups. Furthermore, a linear discriminant analysis effect size (LEfSe) was used to identify microbial taxa significantly enriched in *C. serpentis*-positive versus *C. serpentis*-negative captive snakes. Bacterial, fungal, bacteriophage, nematode, and protozoan taxa were significantly enriched in *C. serpentis*-positive snakes compared with *C. serpentis*-negative captive snakes, based on a linear discriminant analysis (LDA) score ≥ 2.5 and *p* ≤ 0.05. Total taxa species Shannon diversity was consistent between *C. serpentis*-positive and negative captive snakes (p = 0.55) while wild snake samples were significantly more diverse (p = 0.026). Wild snakes also exhibited a significantly increased Shannon diversity of fungi (p = 0.044), protozoa (p = 0.012), and nematodes (p = 0.008) compared to their captive counterparts. This study offers the first in-depth characterization of the cloacal microbiome in reptiles, specifically in EISs, using shotgun metagenomic sequencing. The findings establish a foundation for exploring microbiota–host interactions with implications for reptile health, disease ecology, and conservation management.

## Introduction

The gut microbiome plays a vital role in digestion, immune function, and overall health in vertebrates, including reptiles [1–3]. Previous research investigating the reptilian gut microbiome suggests microbial communities are inherently complex, with their composition known to be influenced by multiple factors such as diet, geography, environment, brumation status, life stage, health condition, intestinal physiochemical characteristics (e.g., pH, oxygen content, viscosity), and anthropogenic disturbance [1,2,4–10]. Given that direct sampling of the gastrointestinal tract is intrusive, cloacal sampling offers a practical alternative method [1,11]. Cloacal samples can serve as a proxy for inferring gut microbiota composition while minimizing stress, discomfort, and the need for invasive or terminal sampling procedures [11–14]. However, it is important to consider that the cloaca’s role as a shared chamber for gastrointestinal, urinary, and reproductive systems, combined with its direct exposure to the external environment, can introduce microbial communities that differ from those within other areas of the gastrointestinal tract. Therefore, interpretations of cloacal microbiome data should acknowledge that these samples capture key gut taxa but may not fully represent the entire gastrointestinal community [1,3,12,13].

The Eastern Indigo Snake (EIS; *Drymarchon couperi*) is a large, non-venomous colubrid native to the southeastern United States and a federally threatened species protected under the Endangered Species Act [15]. While some research has investigated the snake gut microbiome more broadly [16], little research has occurred on snake cloacal microbiomes, with no research specific to EISs. Although captive breeding and reintroduction efforts are key conservation strategies, the long-term health and survival of these snakes in captivity and the wild remain priorities.

EISs present a unique opportunity for investigating cloacal microbiome composition. As primarily ophiophagous predators in the wild, dietary specialization may result in microbial communities that differ from those of ecologically related generalist snakes [17]. EISs are traditionally fed rodents or avian prey in a captive setting, although some institutions and herpetoculturists may also provide reptilian, amphibian, or fish prey items. The influence of prey-associated microbial flora on EIS cloacal microbial communities remains uncertain given their specialized wild diet and mixed feeding practices (live vs. frozen-thawed) in captivity. Recently, an alternative sausage diet was created that mimics the nutritional profile of EISs [18]. One way to determine whether captive diets alter the cloacal microbiome compared to wild snakes would be a direct comparison of both, which to date has not occurred. Understanding dichotomies between wild and captive bred snakes may contribute to the survival of headstarted snakes. If critical cloacal microbiome taxa are absent in captive headstarted snakes, outcomes such as failure to thrive or death may occur.

*Cryptosporidium serpentis* (*C. serpentis*), a protozoan parasite, is a significant health threat in captive snakes, leading to chronic weight loss, gastrointestinal dysfunction, and mortality [19]. Although *C. serpentis* infection and pathology occur exclusively within the gastrointestinal tract, the relationship between cryptosporidiosis and the cloacal microbiome has not been characterized. Cryptosporidiosis often alters the anatomy of the gastric lining in *C. serpentis* infections and the intestinal lining in *Cryptosporidium varanii* (*C. varanii*) infections. When the gut lining physically changes, microbiome niches may change, resulting in possible increase, decrease, and/or extirpation of important microbiota. These shifts likely disrupt microbial community equilibrium and could lead to dysbiosis, as observed in other host species infected with *Cryptosporidium parvum* (*C. parvum*) [20]. It is well known that these microbial communities are directly associated with digestive efficiency [9,16,17].

Clinical signs attributed to gastric cryptosporidiosis (*C. serpentis*) include regurgitation, diarrhea, and decreased fitness over time. Regurgitation occurs from a decreased gastric lumen secondary to marked mucosal hypertrophy and inflammation. The narrowed lumen impedes ingesta resulting in regurgitation. Decrease in fitness is allied to a lack of nutrient absorption due to infection. Diarrhea has been linked to gastrointestinal tract inefficiencies and inflammation [19]. Whether these symptoms are driven by shifts in microbial community composition, anatomical dysfunction, or both remain unknown. Recent advances in *C. serpentis* genomic research have provided the first published metagenome-assembled genome (MAG) of this parasite recovered from EISs [21]. These findings suggest that metagenomic approaches may be useful for assessing associations between *C. serpentis* infection and cloacal microbiome composition, even though the parasite resides in the stomach.

Despite the ecological and conservation importance of reptiles, their cloacal microbiome remains understudied [16,22]. The objective of our study was to compare the cloacal microbiome composition among three groups of EISs: wild individuals, captive *C. serpentis*-negative individuals, and captive *C. serpentis*-positive individuals. We hypothesized that cloacal microbiome composition would vary among the study groups, with each group harboring a distinct microbial community. Specifically, we predicted that *C. serpentis* infection in captive snakes would be associated with significant alterations in the cloacal microbiome, whereas wild snakes would possess a more diverse microbiome than captive snakes, owing to their less controlled environments and diets. By establishing a baseline understanding of the EIS cloacal microbiome, we can better evaluate the effects that captivity, diet, and disease have on this federally threatened species, which is essential for developing prudent conservation programs and management strategies.

## Materials and methods

### Animal selection

#### Captive snakes.

This study was a non-terminal animal study conducted with approval of the Central Florida Zoo & Botanical Gardens’ Research Committee Project #2025-01 and USFWS Permit ES26554C-3. All procedures were minimally invasive and limited to cloacal swab collection, therefore no anesthesia or analgesia was administered. No snakes were euthanized as part of this study. To minimize stress and discomfort, snakes were manually restrained for no more than 10 minutes to safely obtain cloacal swab samples. All handling was performed by trained personnel. Captive snakes were returned to their enclosures, and wild snakes were released at the capture site following sample collection.

As part of a reintroduction program, sixty EISs were housed in captivity according to guidelines set by the Association of Zoos and Aquariums [23]. Briefly, each snake was housed individually in an 18.4 cm x 66.7 cm x 83.8 cm polyvinylchloride drawer and rack system (ARS, Indianapolis, IN, USA) with newsprint substrate within a dedicated room kept at 25.5°C. A thermal gradient was not provided, and each enclosure had a polycarbonate window on one side. Lighting was available from the room’s overhead fluorescent lights and indirect sunlight through the room’s glass window, which was shaded by an outside awning. The fluorescent lights were on for eight hours a day, while the indirect sunlight allowed for a natural seasonal photoperiod. Artificial ultraviolet light was not provided. A diet of frozen-thawed prey items was offered twice weekly, rotating between rats (*Rattus norvegicus*), mice (*Mus musculus*), domestic chicken chicks (*Gallus domesticus*), Japanese quail chicks (*Coturnix japonica*), capelin (*Mallotus villosus*), rainbow trout (*Oncorhynchus mykiss*), and American bullfrog legs (*Lithobates catesbeianus*).

The 60 captive EISs were divided into two groups. The first group consisted of 20 adult male EISs and 10 adult female EISs that had tested negative for *C. serpentis*. The second group consisted of 19 adult male EISs and 11 adult female EISs that had tested positive for *C. serpentis*. The status of *C. serpentis* was determined by probe hybridization qPCR on a gastric lavage sample collected three days after a meal and confirmed with histologic and qPCR analyses of gastric mucosa collected via gastroscopy [24]. The two groups were housed in separate buildings and had no contact with each other.

#### Wild snakes.

Twenty free ranging, wild EISs were opportunistically sampled. One adult male EIS and one adult female EIS were sampled from southwestern Palm Beach County, Florida. One adult male EIS and two adult female EISs were sampled from northwestern Hendry County, Florida. The remaining snakes were collected from a private reserve in one of the reintroduction release sites in Liberty County, Florida and consisted of four adult male EISs and eleven female EISs. Most of these headstarted snakes had been in the wild less than one year (median 285 days; range 230–986 days).

### Sampling

Cloacal samples were collected from both the captive and wild EISs in the same manner. First, the skin around the cloacal opening was wiped clean with 70% isopropyl alcohol-soaked gauze (alcohol prep pads, McKesson Medical-Surgical Inc., Richmond, VA, USA). Once the skin was dry, a sterile HydraFlock® swab (sterile collection swab, Zymo Research Corp. Cat. No. C1100-80, Irvine CA, USA) was inserted roughly 2 cm and rotated within the cloacal lumen five times. The cloacal swabs were then placed in a microbial DNA preservative buffer (DNA/RNA Shield TM, Zymo Research Corp. Cat. No. R1108, Irvine, CA, USA), maintained in freezer storage at approximately –16.5 °C for 8–12 weeks, and sent to MiDOG LLC for DNA extraction and sequencing.

### Metagenomics analysis methods

Genomic DNA (gDNA) was extracted and purified using a commercial kit (ZymoBIOMICS^®^ DNA Microprep Kit (D4301, Zymo Research, Irvine, CA). gDNA samples were profiled with shotgun metagenomic sequencing. Sequencing libraries were prepared with the Illumina DNA Prep Kit (Illumina, San Diego, CA) following the manufacturers protocol using 10-base-pair (bp) unique dual indexes. All libraries were quantified with Qubit (Thermo Fisher Scientific) and then pooled together by equal abundance. The final pool was quantified using quantitative Polymerase Chain Reaction (qPCR). The gDNA was sequenced using the Illumina Novaseq X platform, generating 151-bp paired-end reads. The library produced 20 million (M) paired-end reads per sample, which were trimmed with Trimmomatic v.0.33 [25] to remove low quality fractions and adaptors. Quality trimming was performed on the reads by a sliding window with 6-bp window size and a quality cutoff of 20. Reads with a remaining size lower than 70-bp were removed. Low-diversity reads were detected and removed with sdust v.0.1 (GitHub - lh3/sdust: Symmetric DUST for finding low-complexity regions in DNA sequences). Reads that survived quality trimming were mapped against the genome of *Drymarchon corais* (GCA_043091225.1) using Burrows-Wheeler Alignment (BWA) v.0.7.18 [26] and removed from the pool to eliminate host sequences. The microbial community composition was profiled with Sourmash v 4.8.11 [27] using the k = 51 and scaled = 1000 options to build k-mer signatures from the FASTQ files. These signatures were searched against internally decontaminated databases of publicly available genomes from GTDB and NCBI. These databases included bacterial (*n* = 64,792), fungal (*n* = 3,274), DNA viral (*n* = 23,177), protozoan (*n* = 485), and metazoan (*n* = 412; Nematoda and Platyhelminthes only) genomes, where *n* reflects the number of unique species represented, not the total number of genome assemblies. The Sourmash results were filtered with a coverage threshold of at least 5 kilobase pairs (kbp) to eliminate false positives. The filtered Sourmash results were combined and converted to a phyloseq object for downstream analysis using the R package sourmashconsumr v 0.1.0 [28]. Taxa abundances were calculated using the abundance weighted number of unique k-mers, 51-bp-long DNA sequences derived from the sequencing data that are unique to each taxon in the databases.

Statistical analyses were conducted with R v.4.4.1 [29] using the package PHYLOSEQ v 1.50.0 [30]. Samples were divided into three groups for comparative microbiome analysis: captive snakes with *C. serpentis* infection (Positive, n = 30), captive snakes without *C. serpentis* infection (Negative, n = 30), and wild snakes (Wild, n = 20). The relative abundances of bacteria, fungi, protozoa, bacteriophages, and nematodes were recorded across the three groups of cloacal swabs. Alpha diversity metrics were calculated using the Shannon index and number of observed taxa from each group. Pairwise Wilcoxon tests were performed between *C. serpentis*-positive and *C. serpentis*-negative snakes, and between *C. serpentis*-positive and wild snakes, to test for significant differences in total microbial alpha diversity. Beta-diversity was calculated using Bray-Curtis dissimilarity and Permutational Multivariate Analysis of Variance (PERMANOVA) using adonis2 from the vegan v.2.6.10 R package [31] with 999 permutations. Microbial community structure was analyzed using a Canonical Analysis of Principal Coordinates (CAP) to examine the influence of *C. serpentis* on the cloacal microbiome composition. A linear regression analysis was performed to test possible effects of ambient temperature and snake age on microbial Shannon diversity.

Wild snakes were removed from the dataset and a linear discriminant analysis effect size (LEfSe) test was performed using the R package microbiomeMarker v.1.12.2 [32] to identify microbial taxa that were significantly enriched in *C. serpentis*-positive vs. *C. serpentis*-negative captive snakes. Bacterial, fungal, bacteriophage, nematode and protozoan species were determined to be significantly enriched by a linear discriminant analysis (LDA) score ≥ 2.5 and P-value ≤ 0.05. DESEQ2 v.1.50.2 [33] was used to further statistically confirm differential abundance of taxa between *C. serpentis*-positive and *C. serpentis*-negative snakes using absolute log2fold change > 1.5 and P-value ≤ 0.05, as it models count data directly, accounts for variance-mean dependence, and performs robust statistical testing with multiple testing correction.

To assess the functional differences in cloacal microbiomes, HUMAnN3 v3.6 [34] was used to quantify the abundances of bacterial gene families and metabolic pathways. The trimmed, host-depleted reads were concatenated and aligned against the UniRef90 and ChocoPhlAn databases using default parameters. Significant differences in pathway abundances between wild, *C. serpentis*-positive, and *C. serpentis*-negative snakes were tested for using MaAsLin2 v.1.16.0 R package [35]. Wild snakes were again removed from the study to test for significant pathway differences between captive *C. serpentis*-positive and negative snakes.

## Results

**Fig 1:**
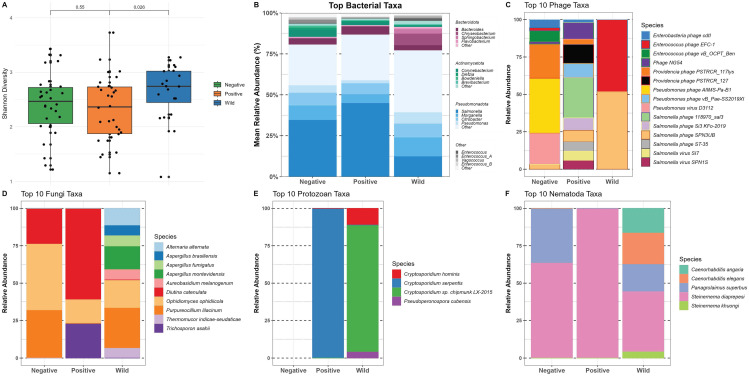
Diverse microbial taxa from multiple kingdoms are observed in the cloacal samples. (A) A boxplot comparing Shannon diversity is presented for captive *C. serpentis*-positive (n = 30), captive *C. serpentis*-negative (n = 30), and wild snakes (n = 20). P-values are displayed above the boxes to indicate statistically significant differences among the three groups. P-values < 0.05 indicate significant differences between the respective groups. (B–F) Bar plots show the relative abundance of the most prevalent taxa identified in this study across five major viral and microbial taxonomic groups: (B) bacteria, (C) bacteriophages, (D) fungi, (E) protozoa, and (F) nematodes. The percentages represent the relative abundance of each taxon within the respective sample group and its corresponding taxonomy group.

Pairwise Wilcoxon tests based on Shannon diversity and Bray-Curtis beta diversity using adonis2 showed no significant differences between wild and headstarted snakes (p = 0.5 and p = 0.073, respectively) (S1 Fig). Therefore, for all subsequent analyses, wild and headstarted snakes were consolidated into a single ‘Wild’ group. The total classified microbiomial Shannon diversity was consistent between *C. serpentis*-positive and negative captive snakes (p = 0.55) while the wild snakes exhibited significantly greater Shannon diversity (p = 0.026) (**Fig 1A**). Wild snakes exhibited a significant increased Shannon diversity of fungi (p = 0.044), protozoa (p = 0.012), and nematodes (p = 0.008) but no significant difference in classified bacteria (p = 0.20) or bacteriophages (p = 0.39) compared to their captive counterparts. Within the classified fraction, the cloacal microbiome of the snakes was dominated by the phylum Pseudomonadota, especially *Salmonella* species, while Actinomycetota, Bacteroidota, and Bacillota were found at relatively low abundance (**Fig 1B**). Similarly, the viral communities within the cloacal samples were dominated by *Salmonella* and *Pseudomonas* bacteriophages (**Fig 1C**). Only two bacteriophage species *Enterococcus phage EFC-1* and *Salmonella phage SPN3UB* were identified in cloacal swabs from wild snakes while captive snakes exhibited a greater diversity of identified bacteriophage species as a whole. The cloacal swabs of wild snakes contained two fungal genera, *Aspergillus* and *Alternaria*, which were completely absent in captive snakes (**Fig 1D**). Conversely *Diutina catenulate*, *Purpeocillium lilacinum*, and the snake fungal pathogen *Ophidiomyces ophiodiicola* were conserved across captive and wild snakes. The Basidiomycete yeast fungal pathogen, *Trichosporon asahii,* was found in only captive snakes infected with *C. serpentis* (**Fig 1E**). Four protozoan species were observed across the snake samples. *C. serpentis* was identified in the metagenomic data of two of the 30 snakes that tested positive for *C. serpentis* and was completely undetected in all negative and wild snakes. Two additional species of *Cryptosporidium*, *Cryptosporidium* sp. chipmunk LX-2015 and *Cryptosporidium hominis*, were only detected in wild snakes. Of the five nematode species observed in this study, one species, *Steinernema diaprepesi*, was found in samples from all three groups of snakes (**Fig 1F**). No nematode taxa were unique to *C. serpentis*-positive snakes. Despite these findings, ~ 96–99% of the metagenomic k-mers remained unclassified across samples (**S2 Fig**), highlighting the substantial proportion of undescribed microbial diversity present in the EIS cloacal microbiome.

**Fig 2:**
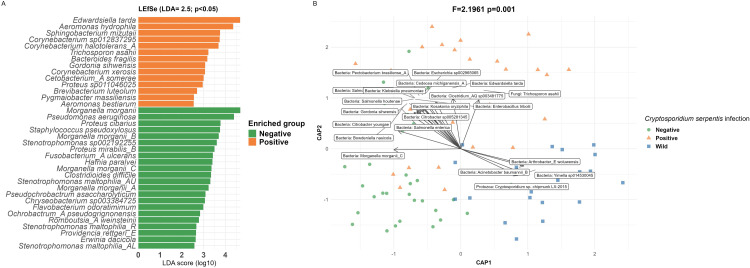
Distinct microbial taxa are enriched in wild, *C. serpentis*-negative, and *C. serpentis*-positive snakes. (A) Bar plot showing microbial species significantly enriched in captive *C. serpentis*-positive snakes (orange) and captive *C. serpentis*-negative snakes (green), based on LDA scores > 2.0 and P-values < 0.05. The taxa corresponding to orange bars represent the taxa enriched in *C. serpentis*-positive snakes while green bars correspond to *C. serpentis*-negative snakes. The x-axis reflects the LDA score, with higher LDA scores reflecting a stronger association between the microbial species and the snake group. (B) Constrained Analysis of Principal Coordinates (CAP) based on Bray-Curtis dissimilarity illustrating differences in bacterial, fungal, protozoan, nematode, and bacteriophage communities among wild, captive *C. serpentis*-negative, and captive *C. serpentis*-positive snakes. Each point represents an individual sample, colored and shaped by snake group. Green circles represent captive *C. serpentis*-negative snakes, orange triangles are captive *C*. *serpentis*-positive snakes, and blue squares are wild snakes. Arrows indicate microbial taxa significantly associated with each group; arrow length and direction reflect the strength and gradient of association within the ordination space. For example, longer arrows pointing toward the wild group of snakes represent a stronger association of the indicated taxon to wild snakes. The F-score and P-values of a Bray-Curtis dissimilarity test are shown above the plot, indicating the significant difference in beta diversity between the three snake groups.

The LEfSe analysis confirmed several classified taxa that are significantly enriched in *C. serpentis*-positive snakes including *Aeromonas hydrophila*, *Edwardsiella tarda*, and *Trichosporon asahii* (**Fig 2A**). The beta diversity analysis based on Bray-Curtis distance indicated significant, yet weak, microbiome differences between *C. serpentis*-positive, *C. serpentis*-negative, and wild snakes (R^2^ = 0.0738, F = 2.1961, p < 0.001) (**Fig 2B**) but not between *C. serpentis*-positive and negative snakes alone. Taxa vectors overlaid on the ordination plot revealed that *Cryptosporidium* sp. chipmunk LX-2015 was significantly associated with wild snakes. In contrast, *Trichosporon asahii* and *Edwardsiella tarda* were strongly associated with captive *C. serpentis*-positive snakes. Of these taxa, *Trichosporon asahii* (log2fold = 10.733, p = 2.25e-5), and *Edwardsiella tarda* (log2fold = 5.24, p = 9.02e-3) were confirmed to be significantly enriched in *C. serpentis*-positive snakes based on DESEQ2 analysis.

**Fig 3:**
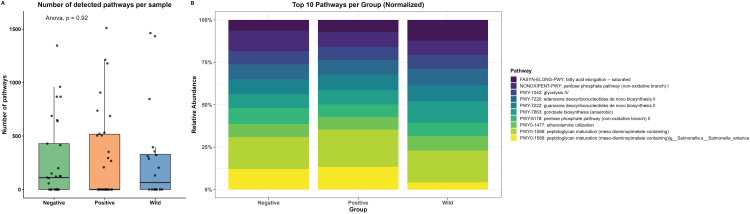
No significant differences in gene pathway abundances were detected between wild, *C. serpentis*-negative, and *C. serpentis*-positive snakes. (A) Boxplots based on the number of described microbial pathways present in captive *C. serpentis*-positive snakes (orange), captive *C. serpentis*-negative (green), and wild snakes (blue). Each point represents an individual sample, and box boundaries indicate the interquartile range with the median shown as a horizontal line within the box. The P-value representing the ANOVA significance test is shown above the plot. The points represent the number of pathways per sample. (B) Stacked bar plots illustrating the relative abundance and identities of the top 10 most abundant pathways (excluding unmapped and integrated pathways) across the three groups of snakes. Each bar represents a group, and the segments of the bars correspond to the individual pathways. Colors denote the distinct pathways identities, allowing comparison of pathway composition and relative contribution across groups.

The HUMAnN3 analysis was unable to map the majority of the reads in each sample (91–99.7% unmapped). Furthermore, 0.53–7.9% of the remaining reads per sample were unintegrated. The top 10 most abundant integrated pathways encoded functions for peptidoglycan synthesis, and ethanolamine utilization (**Fig 3B**). MaAsLin2 analysis revealed no significant differences in any gene pathway abundance between captive *C. serpentis*-positive and negative snakes (p = 0.96). Additionally, no significant differences in the number of pathways or gene family abundances were identified when including wild snakes (p = 0.92) (**Fig 3A**).

## Discussion

Our study investigated differences in the cloacal microbiome of EISs, focusing on comparisons between wild individuals and captive snakes that were either negative or positive for *C. serpentis*. To our knowledge, this represents the first application of shotgun metagenomic sequencing to characterize the ophidian cloacal microbiome. Previous gut and cloacal microbiome research in reptiles has primarily focused on using amplicon-based methodologies (16S rRNA gene sequencing or internal transcribed spacer (ITS) sequencing) [36], as shotgun metagenomics has only been used in a limited capacity to characterize fecal, rather than cloacal, microbiota in snakes [9,37]. Genomic data is increasingly being incorporated into conservation decisions and the development of management plans for imperiled species [38,39]. The development of sequencing techniques has enabled managers to rapidly develop genome scale data for species to monitor genetic diversity not only in wild populations, but also breeding colonies. We have demonstrated that shotgun metagenomic sequencing provides valuable insight into the cloacal microbiome. This approach may also aid in identifying microbial associations relevant to disease status and overall snake health, as shifts in microbial diversity and community composition may reflect dysbiosis and alterations in immune function. Furthermore, our study is the first to reveal how the cloacal microbiome of snakes shifts following release from captive headstart programs into the wild, while concurrently identifying the unique community of wild-associated microbial species linked to EISs. The shift in community composition seen in wild populations of EISs shows that their cloacal microbiome is dynamic, and that microbiome compositional biases from captive rearing can shift post-reintroduction. In previous studies, cloacal microbiome samples from Fiji crested iguanas (*Brachylophus vitiensis*) and Texas horned lizards (*Phrynosoma cornutum*) that were headstarted in captivity required approximately two months after release for their bacterial communities to become indistinguishable from those of wild conspecifics [4,22]. In addition to traditional genome scale data, metagenomic data should be incorporated into management plans to better understand how captivity, health status, and ecological factors may alter the microbiome of target species. In turn, these data can then be used to inform management practices, improve captive husbandry standards, and support conservation initiatives aimed at protecting wild populations.

### Bacteria

In our study, bacteria represented the dominant kingdom in the cloacal microbiome of EISs. Previous microbiome studies have identified Pseudomonadota (formerly Proteobacteria), Bacteroidota (formerly Bacteroidetes), and Bacillota (formerly Firmicutes) as major core phyla associated with the reptilian gastrointestinal tract [2]. Pseudomonadota was the most abundant bacterial phylum detected in our study, consistent with findings from other reptile gut and/or cloacal microbiome studies utilizing 16S rRNA amplicon sequencing [1,3,6,9,12,22,40]. As a facultative anaerobe, Pseudomonadota is thought to aid in the breakdown of complex sugars, promote nutrient absorption, and provide a protective role in immune responses against infection or inflammation [16,37,41]. Aerobic conditions in the cloaca may lead to this environment being dominated by Pseudomonadota [3,42]. However, a persistent overrepresentation of Pseudomonadota may indicate dysbiosis or underlying gastrointestinal disease [1]. Notably, other studies have shown that the gut bacterial microbiota of some snake species, such as captive Burmese Pythons, are dominated by both Bacteroidota and Bacillota phyla [43]. Our study detected only a small proportion of microbes from the phylum Bacillota. These findings suggest that the cloacal microbiome of EISs may harbor distinct microbial communities upstream in the gastrointestinal tract, which could differ from other snake species.

In our LEfSe analysis, *Aeromonas hydrophila* (*A. hydrophila*) and *Edwardsiella tarda* (*E. tarda*) were strongly enriched in captive *C. serpentis*-positive snakes. This result suggests the presence of potential bacterial co-morbidities that may interact with *C. serpentis* under captive conditions. Both *A. hydrophila* and *E. tarda* are frequently implicated as fish pathogens and are widely acknowledged as zoonotic organisms with enteropathogenic potential in humans [44,45]. *Aeromonas* spp., including *A. hydrophila*, are known to cause gastroenteritis, wound infections, and bacteremia with virulence factors that facilitate intestinal colonization and invasion [46–48]. Typically, *E. tarda* presents with human gastroenteritis and in severe cases can lead to bacteremia [49,50]. Although all captive snakes were periodically fed prey items originating from aquatic environments that may serve as sources of *Aeromonas* and *Edwardsiella* spp., including capelin (*Mallotus villosus*), rainbow trout (*Oncorhynchus mykiss*), and American bullfrog legs (*Lithobates catesbeianus*), the absence of enrichment in *C. serpentis*-negative snakes suggests that dietary exposure alone is unlikely to explain the observed association.

Experimental work further supports the possibility of synergistic interactions between *Aeromonas* and *Cryptosporidium*. In a murine coinfection model, *A. hydrophila* and *C. parvum* resulted in mutually prolonged colonization within the spleen and intestines [51]. This data suggests that *Aeromonas* and *Cryptosporidium* may enhance their pathogenic potential and create a more significant infectious burden than either organism would cause alone. The detection of *E. tarda* alongside *A. hydrophila* in *C. serpentis*-positive snakes may reflect gastrointestinal dysbiosis associated with captivity and promote conditions where multiple opportunistic pathogens can coexist and interact, thereby exacerbating the disease process and posing potential zoonotic risks [20,52].

In the present study, there was a significant difference in microbial beta diversity between all three study groups. Similarly, differences in beta diversity between wild and captive populations have been reported across multiple reptile species [4,22,42,53]. In our findings, wild EISs exhibited greater Shannon diversity of cloacal microbiota taxa compared to their captive counterparts. Greater species Shannon diversity observed in wild snakes may result from increased environmental microbial exposure, a more varied diet, and other environmental factors (temperature, humidity, brumation etc.). It is important to note however that environmental microbial communities were not evaluated in this study. Anthropogenic influences, such as human disturbance, may also contribute to microbiome differences between groups, as suggested in *Sceloporus* lizards [7]. Future studies that analyze environmental microbiota will be important for understanding how habitat, captivity, and host health interact to shape microbial community composition.

### Fungi

Research on the fungal component of the reptilian gut microbiome remains limited, and studies of the cloacal microbiome are even more sparse, with most work focusing on yeasts as potential pathogens and their role as environmental reservoirs using the internal transcribed spacer (ITS) region [54–56]. In our study, LEfSe analysis showed a significant enrichment of *Trichosporon asahii* (*T. asahii*) in the cloacal microbiome of *C. serpentis*-positive snakes. Notably, *T. asahii* occurred exclusively in captive *C. serpentis*-positive snakes, suggesting a potential association between both pathogens. Immunocompromised animals are more prone to invasive yeast infections [57], making this association biologically plausible. In EISs, *C. serpentis* infection has been associated with stress-induced immunosuppression [19,58,59], a phenomenon similarly documented in humans with HIV [55,56].

*Trichosporon* yeasts are widespread in soil, can cause life-threatening systemic infections in immunocompromised hosts, and are resistant to multiple antifungal medications [55,56,60,61]. Trichosporonosis in reptiles is most often reported as opportunistic shell infections in chelonians but has also been associated with fatal disseminated disease in Banded Rock Rattlesnakes (*Crotalus lepidus klauberi*), as well as lesions affecting the skin, lungs, gut, liver, kidney, eyes and oral cavity of tortoises, lizards, snakes, and crocodilians [61,62]. In our study, *T. asahii* was restricted to captive *C. serpentis*-positive snakes, likely reflecting pathogen-driven dysbiosis, host immunosuppression, or environmental exposure (e.g., water dishes, humid hides, substrate, etc.). Its absence in wild and *C. serpentis*-negative snakes suggests it may opportunistically colonize disrupted microbial environments. Additionally, *Trichosporon* species have been isolated from 9% of healthy squamates, with molecular analyses identifying *T. asahii*, *T. inkin*, and *T. cutaneum* [62]. These findings suggest *Trichosporon* spp. may have a role as both commensal flora and opportunistic pathogens under stress or immunosuppression.

We also detected *Ophidiomyces ophiodiicola (O. ophiodiicola)*, the agent of snake fungal disease (SFD), in all study groups. SFD is an emerging infectious disease in North American snakes, which causes dermal crusts that can progress to ulceration, invasion of deeper tissues, and fatal systemic infection [63,64]. *O. ophiodiicola* infected Northern Cottonmouths (*Agkistrodon piscivorus*) had a median survival of 90 days and 40% mortality in experimental trials [63]. *O. ophiodiicola* also induces skin microbiome dysbiosis [65], suggesting that gut or cloacal colonization may result in similar pathogen-driven community shifts. Although cloacal sites were disinfected with 70% isopropyl alcohol-soaked gauze prior to sample collection, the possibility of contamination during swabbing cannot be entirely ruled out. More invasive sampling approaches, such as biopsy, may be necessary to definitively confirm its presence within the gastrointestinal tract.

Multiple other opportunistic fungi were detected including *Diutina catenulata*, *Purpureocillium lilacinum*, *Aspergillus* spp., *Alternaria alternata*, and *Aureobasidium melanogenum*. *Diutina catenulata* (formerly *Candida catenulata*), a common yeast in human and animal gut microbiomes, was identified across all study groups [54]. In contrast, *Purpureocillium lilacinum*, a saprophytic fungus and known opportunistic pathogen [66], was detected in captive *C. serpentis*-negative and wild snakes but was rare in *C. serpentis*-positive individuals. Both organisms were likely acquired through diet or environmental exposure [56,67,68], with reduced abundance of *Purpureocillium lilacinum* in infected snakes possibly due to disease-associated microbiome changes. *Aspergillus* spp., *Alternaria alternata*, and *Aureobasidium melanogenum* were detected only in wild snakes, consistent with increased exposure to environmental fungal spores. Their absence in captive EISs may be due to reduced exposure in controlled environments with air filtration systems. Collectively, these findings suggest that the presence and distribution of opportunistic fungi in the cloacal microbiome may be influenced by a combination of environmental exposure, diet, and disease status.

### Protozoa

Only 2 of 30 known *C. serpentis*-positive captive snakes were actively shedding oocysts at the time of cloacal swab sampling. Low-to-intermittent oocyst shedding is well documented in snakes with gastric cryptosporidiosis and complicates prevalence estimates when single time-point cloacal swabs are used [19,69,70]. In addition to intermittent shedding, sampling methodology may also have contributed to the low prevalence in our captive positive samples. Recent comparative work in EISs shows that diagnostic yield for *C. serpentis* varies by specimen type and test. Gastric biopsy outperformed cloacal swabbing and *C. serpentis*-specific probe hybridization qPCR outperformed Hsp70 PCR for detecting low-level or intermittent oocyst shedding [58]. Gastroscopy with targeted gastric biopsy performed three days after a meal remains the most sensitive sample type and *C. serpentis*-specific probe hybridization qPCR paired with histologic analysis of gastric mucosa remains the most sensitive test type for detection of *C. serpentis* [19,71]. Using serial cloacal or gastric swabs and sensitive assays such as *C. serpentis*-specific probe hybridization qPCR to improve detection may be a viable alternative if gastric biopsy is unable to be performed [58].

The restriction of *Cryptosporidium* chipmunk LX-2015 (also called chipmunk genotype I) and *Cryptosporidium hominis* (*C. hominis*) to wild snakes in our dataset suggest environmental spillover rather than an established snake infection. Both taxa are recognized human pathogens, with *C.* chipmunk LX-2015 being confirmed as zoonotic [72,73], and *C. hominis* being the dominant anthroponotic species [74,75]. Their detection in wild snakes could reflect ingestion of infected prey (e.g., small mammals or reptiles), exposure to contaminated surface water, or transient gut passage without gastrointestinal colonization. These results highlight the role of wildlife–environment–human interfaces in potential transmission pathways.

Protozoa were not detected in the negative captive group. However, because *C. serpentis* sheds intermittently and primarily affects the stomach, continued monitoring with repeated samples is recommended for colony management and conservation programs.

### Viruses

The virome analysis revealed the presence of bacteriophages as the only detectable viral group. Notably, wild snakes exhibited a reduced bacteriophage diversity relative to their captive counterparts. One plausible explanation for this observation is that wild snakes may harbor a greater proportion of viral dark matter, which would include novel or uncharacterized viruses that remain undetectable with current reference databases. An additional consideration is that metagenomic sequencing in this study was limited to DNA viruses, thereby excluding RNA viruses from detection and potentially underestimating the overall viral diversity present in our samples.

### Nematodes

Nematode diversity was limited and no reptile-specific pathogens were identified. *Steinernema diaprepesi*, an entomopathogenic nematode, was consistently detected across all groups. This association suggests it may represent an environmental contaminant (possibly from substrate or consumed prey item) rather than a host-associated parasite [76]. Wild snakes harbored the greatest diversity of nematodes, consistent with broader environmental exposure compared to the restricted conditions of captivity.

### Sampling methodology and study limitations

Sampling methodology can influence microbial diversity. Previous studies have shown that microbial composition varies along the gastrointestinal tract [1,12] and aerobic conditions in the cloaca may influence the microbial community present [3,42]. Cloacal swabs, while providing a broad overview of gut bacterial diversity, may not fully represent hindgut communities. For example, in Northern Cottonmouths (*Agkistrodon piscivorus*), nearly 50% of the microbial composition in the cloaca was dominated by Pseudomonadota and reflected similar bacterial diversity in the small intestines [12]. Conversely, fecal samples are often more representative of hindgut communities and may exhibit higher alpha diversity, as observed in sea turtles where Bacillota and Bacteroidota predominated, while Pseudomonadota dominated cloacal microbiomes [3]. In *Sceloporus virgatus* lizards, fecal samples were enriched with Bacillota and Bacteroidota, while cloacal swab samples exhibited a higher abundance of Pseudomonadota [14]. Furthermore, cloacal and fecal sampling methods revealed significant differences between captive and wild Fijian crested iguanas (*Brachylophus vitiensis*), with each sample type exhibiting distinct bacterial community structure and diversity [4]. Given these considerations, future minimally invasive studies on EIS should incorporate fecal sampling alongside cloacal swabs to obtain a more comprehensive view of gut and cloacal microbial diversity and abundance. The combined approach could reveal microbial communities and patterns that might otherwise be underrepresented when relying solely on cloacal swabs.

Several limitations should be considered when interpreting these findings. First, environmental samples such as soil, water, or enclosure surfaces were not collected alongside cloacal swabs. The cloaca is directly exposed to the external environment, making it plausible that environmental microbes may influence the cloacal microbial community. Without environmental sampling, however, the contribution of environmental microbes to the cloacal microbiome in wild and captive EIS remains inferential and warrants further investigation in future studies. However, a study in Texas horned lizards (*Phrynosoma cornutum*) reported that environmental microbes did not significantly contribute to the cloacal microbiome community [22]. Dietary microbiota sampling of captive prey items and evaluating their contribution to the captive EIS cloacal microbiome may also be of benefit [22]. Additionally, a limitation to microbiome analysis using shotgun metagenomic sequencing is the availability of reference genomes to classify DNA reads. While genomic resources for bacteria are relatively robust, the same is not true for the protozoan and metazoan species. For example, the genome for *Cryptosporidium varanii* has not been sequenced, and these samples could not be evaluated for *C. varanii* by this methodology. Similarly, at the time of this writing, there is no published genome of the host *Drymarchon couperi* and the genome of *Drymarchon corais* was used as a substitute to remove host reads from the dataset. The reader should interpret the data with this in mind, but as metagenomic sequencing continues to be a growing field, genomic databases continue to expand and taxonomic resolution will improve. In our study, a substantial portion of sequencing reads could not be taxonomically classified. This underscores the importance of acknowledging and reporting this undescribed DNA, or “dark matter”, in the samples (see supplemental data) to enable future studies and comparisons. The authors encourage similar studies to report detected dark matter in their samples and pursue follow-up experiments using the sequence data deposited with this manuscript.

## Supporting information

S1 Fig There are no significant differences in Shannon diversity or Bray-Curtis beta diversity between wild and headstarted snakes. (**A**) A boxplot is shown comparing the Shannon diversity between wild South Florida snakes (SFL) and headstarted snakes from a private reserve (ABRP). The p-value from the pairwise t-test is shown above the boxplot. (**B**) A PCoA plot is shown comparing the beta-diversity between wild South Florida snakes (SFL) and headstarted snakes from a private reserve (ABRP). The F-score and p-value are shown above the plot.
(DOCX)

S2 Fig The cloacal microbiome of EIS is largely unclassified. A bar plot is shown indicating the abundance-weighted fraction of k-mer signatures from each sample that are classified to each database. The y-axis represents the fraction of k-mers from the sample that align to each database. The gray portions of the bars depict proportions of the samples that are unclassified.
(DOCX)

S3 Fig The classified cloacal microbiome of EIS is dominated by bacteria. A bar plot is shown indicating the abundance-weighted fraction of k-mer signatures from each sample that are classified to the kingdoms Bacteria, Fungi, Virus, Protozoa, and Metazoa. The y-axis represents the fraction of k-mers from the sample that align to each kingdom.
(DOCX)

S4 Fig The classified cloacal microbiome Shannon diversity is not correlated with temperate, age, or sex of the snake. Two linear regression plots are shown indicating the Shannon Diversity of the classified cloacal microbiomes compared with (**A**) snake age in days and (**B**) temperature in Farenheit when the sample was collected. P-values are labeled in each plot. Sample points in the figures are shaped by snake sex.
(DOCX)

S5 Fig *Cryptosporidium serpentis* is the only protist observed in captive snakes while the wild snakes contain more eukaryotic diversity. (Top) A Venn diagram is shown depicting the number of unique protozoan species observed between the three groups of snakes in this study. The table below the diagram shows the kingdom and species of the four protozoan taxa and the groups of snakes they were observed in.
(DOCX)

## References

[1] Tang W, Zhu G, Shi Q, Yang S, Ma T, Mishra SK, et al. Characterizing the microbiota in gastrointestinal tract segments of *Rhabdophis subminiatus*: dynamic changes and functional predictions. Microbiologyopen. 2019;8(7):e00789. doi: 10.1002/mbo3.789 30848054 PMC6612554

[2] Siddiqui R, Maciver SK, Khan NA. Gut microbiome-immune system interaction in reptiles. J Appl Microbiol. 2022;132(4):2558–71. doi: 10.1111/jam.15438 34984778

[3] Forbes Z, Scro A, Patel S, Dourdeville K, Prescott R, Smolowitz R. Fecal and cloacal microbiomes of cold-stunned loggerhead *Caretta caretta*, Kemp’s ridley *Lepidochelys kempii*, and green sea turtles *Chelonia mydas*. Endanger Species Res. 2023;50:93–105. doi: 10.3354/esr01220

[4] Eliades SJ, Brown JC, Colston TJ, Fisher RN, Niukula JB, Gray K, et al. Gut microbial ecology of the Critically Endangered Fijian crested iguana (*Brachylophus vitiensis*): effects of captivity status and host reintroduction on endogenous microbiomes. Ecol Evol. 2021;11(9):4731–43. doi: 10.1002/ece3.7373 33976843 PMC8093715

[5] Trevelline BK, Fontaine SS, Hartup BK, Kohl KD. Conservation biology needs a microbial renaissance: a call for the consideration of host-associated microbiota in wildlife management practices. Proc Biol Sci. 2019;286(1895):20182448. doi: 10.1098/rspb.2018.2448 30963956 PMC6364583

[6] Jiang HY, Ma JE, Li J, Zhang XJ, Li LM, He N, et al. Diets alter the gut microbiome of crocodile lizards. Front Microbiol. 2017;8:2073. doi: 10.3389/fmicb.2017.02073 29118742 PMC5660983

[7] Bunker ME, Weiss SL. Cloacal microbiomes of sympatric and allopatric Sceloporus lizards vary with environment and host relatedness. PLoS One. 2022;17(12):e0279288. doi: 10.1371/journal.pone.0279288 36548265 PMC9779040

[8] McNally KL, Innis CJ, Kennedy A, Bowen JL. Characterization of oral and cloacal microbial communities in cold-stunned Kemp’s ridley sea turtles (*Lepidochelys kempii*) during the time course of rehabilitation. PLoS One. 2021;16(5):e0252086. doi: 10.1371/journal.pone.0252086 34043685 PMC8159006

[9] Cong X, Liu X, Zhou D, Xu Y, Liu J, Tong F. Characterization and comparison of the fecal bacterial microbiota in Red Back Pine Root Snake (*Oligodon formosanus*) and Chinese Slug-Eating Snake (*Pareas chinensis*). Front Microbiol. 2025;16:1575405. doi: 10.3389/fmicb.2025.1575405 40309103 PMC12040955

[10] Ferreira-Machado E, Navas-Suárez PE, Ervedosa TB, Figueiredo KB, de Carvalho ACSR, Takahashi JPF, et al. Infections by entomopathogenic fungi in common green iguanas (*Iguana iguana*) in captivity in Brazil. J Comp Pathol. 2023;201:16–22. doi: 10.1016/j.jcpa.2022.12.006 36646035

[11] Kuschke SG. What lives on and in the sea turtle? A literature review of sea turtle bacterial microbiota. Anim Microbiome. 2022;4(1):52. doi: 10.1186/s42523-022-00202-y 36076281 PMC9461204

[12] Colston TJ, Noonan BP, Jackson CR. Phylogenetic analysis of bacterial communities in different regions of the gastrointestinal tract of agkistrodon piscivorus, the cottonmouth Snake. PLoS One. 2015;10(6):e0128793. doi: 10.1371/journal.pone.0128793 26039313 PMC4454441

[13] Dallas JW, Meshaka WE Jr, Zeglin L, Warne RW. Taxonomy, not locality, influences the cloacal microbiota of two nearctic colubrids: a preliminary analysis. Mol Biol Rep. 2021;48(9):6435–42. doi: 10.1007/s11033-021-06645-x 34403035

[14] Bunker ME, Martin MO, Weiss SL. Recovered microbiome of an oviparous lizard differs across gut and reproductive tissues, cloacal swabs, and faeces. Mol Ecol Resour. 2022;22(5):1693–705. doi: 10.1111/1755-0998.13573 34894079

[15] Hammerson GA. Drymarchon couperi. 2007. Available from: https://www.iucnredlist.org/species/63773/12714602

[16] Colston TJ, Jackson CR. Microbiome evolution along divergent branches of the vertebrate tree of life: what is known and unknown. Mol Ecol. 2016;25(16):3776–800. doi: 10.1111/mec.13730 27297628

[17] Hoffbeck C, Middleton DMRL, Nelson NJ, Taylor MW. 16S rRNA gene-based meta-analysis of the reptile gut microbiota reveals environmental effects, host influences and a limited core microbiota. Mol Ecol. 2023;32(22):6044–58. doi: 10.1111/mec.17153 37795930

[18] Jackson PR, Bogan JE Jr, Dierenfeld ES, Loughman ZJ. Evaluation of nutritional and health status in Captive Eastern Indigo Snakes (*Drymarchon couperi*) in response to formulated sausage diet. Animals (Basel). 2024;14(22):3324. doi: 10.3390/ani14223324 39595376 PMC11591334

[19] Bogan JE. Gastric Cryptosporidiosis in Snakes, a Review. J Herpetol Med Surg. 2019;29(3–4):71–86. doi: 10.5818/19-05-201.1

[20] Mammeri M, Obregón DA, Chevillot A, Polack B, Julien C, Pollet T, et al. *Cryptosporidium parvum* infection depletes butyrate producer bacteria in goat kid microbiome. Front Microbiol. 2020;11:548737. doi: 10.3389/fmicb.2020.548737 33178145 PMC7596689

[21] Yacoub MN, Krumbeck JA, Bogan JE Jr, Mason AK. First metagenome-assembled genome of *Cryptosporidium serpentis* from Drymarchon couperi gastric lavage. Microbiol Resour Announc. 2025;14(9):e0034225. doi: 10.1128/mra.00342-25 40741758 PMC12424359

[22] Forehand CR, Smith SN, Nielsen F, Bauer B, Watters JL, Moody RW, et al. Comparative assessment of Texas horned lizard (*Phrynosoma cornutum*) gut microbiome diversity and composition throughout transition from captivity to wild. Front Microbiomes. 2025;4:1601442. doi: 10.3389/frmbi.2025.1601442 41852402 PMC12993635

[23] Antonio F. Eastern indigo snake (*Drymarchon couperi*) care manual. Silver Spring, MD, USA: Association of Zoos and Aquariums; 2011. pp. 5–42.

[24] Bogan JE Jr, Wellehan JFX Jr, Garner MM, Childress AL, Jackson B. Evaluation of a probe hybridization quantitative polymerase chain reaction assay for *Cryptosporidium serpentis* in eastern indigo snakes (*Drymarchon couperi*). Parasitol Res. 2022;121(12):3523–7. doi: 10.1007/s00436-022-07676-4 36171408 PMC9653349

[25] Bolger AM, Lohse M, Usadel B. Trimmomatic: a flexible trimmer for Illumina sequence data. Bioinformatics. 2014;30(15):2114–20. doi: 10.1093/bioinformatics/btu170 24695404 PMC4103590

[26] Li H, Durbin R. Fast and accurate short read alignment with Burrows-Wheeler transform. Bioinformatics. 2009;25(14):1754–60. doi: 10.1093/bioinformatics/btp324 19451168 PMC2705234

[27] Irber L, Pierce-Ward NT, Abuelanin M, Alexander H, Anant A, Barve K, et al. sourmash v4: A multitool to quickly search, compare, and analyze genomic and metagenomic data sets. J Open Source Softw. 2024;9(98):6830. doi: 10.21105/joss.06830

[28] Chou S, Reiter T. A new R package, sourmashconsumr, for analyzing and visualizing the outputs of sourmash. Arcadia Sci. 2023. doi: 10.57844/arcadia-1896-ke33

[29] R Core Team. R: A language and environment for statistical computing. Vienna, Austria: R Foundation for Statistical Computing. 2021. Available from: https://www.R-project.org/

[30] McMurdie PJ, Holmes S. phyloseq: an R package for reproducible interactive analysis and graphics of microbiome census data. PLoS One. 2013;8(4):e61217. doi: 10.1371/journal.pone.0061217 23630581 PMC3632530

[31] Oksanen J, Simpson G, Blanchet F, Kindt R, Legendre P, Minchin P, et al. vegan: Community Ecology Package. 2025. Available from: https://CRAN.R-project.org/package=vegan

[32] Cao Y, Dong Q, Wang D, Zhang P, Liu Y, Niu C. microbiomeMarker: an R/Bioconductor package for microbiome marker identification and visualization. Bioinformatics. 2022;38(16):4027–9. doi: 10.1093/bioinformatics/btac438 35771644

[33] Love MI, Huber W, Anders S. Moderated estimation of fold change and dispersion for RNA-seq data with DESeq2. Genome Biol. 2014;15(12):550. doi: 10.1186/s13059-014-0550-8 25516281 PMC4302049

[34] Beghini F, McIver LJ, Blanco-Míguez A, Dubois L, Asnicar F, Maharjan S, et al. Integrating taxonomic, functional, and strain-level profiling of diverse microbial communities with bioBakery 3. Elife. 2021;10:e65088. doi: 10.7554/eLife.65088 33944776 PMC8096432

[35] Mallick H, Rahnavard A, McIver LJ, Ma S, Zhang Y, Nguyen LH, et al. Multivariable association discovery in population-scale meta-omics studies. PLoS Comput Biol. 2021;17(11):e1009442. doi: 10.1371/journal.pcbi.1009442 34784344 PMC8714082

[36] Vargas-Gastélum L, Romer AS, Ghotbi M, Dallas JW, Alexander NR, Moe KC, et al. Herptile gut microbiomes: a natural system to study multi-kingdom interactions between filamentous fungi and bacteria. mSphere. 2024;9(3):e0047523. doi: 10.1128/msphere.00475-23 38349154 PMC10964425

[37] Hu X, Yang L, Zhang Y, Yang M, Li J, Fan Y, et al. Fecal and oral microbiome analysis of snakes from China reveals a novel natural emerging disease reservoir. Front Microbiol. 2024;14:1339188. doi: 10.3389/fmicb.2023.1339188 38274764 PMC10808610

[38] Hoban S, da Silva JM, Mastretta‐Yanes A, Grueber CE, Heuertz M, Hunter ME. Monitoring status and trends in genetic diversity for the convention on biological diversity: an ongoing assessment of genetic indicators in nine countries. Conserv Lett. 2023;16:e12953. doi: 10.1111/conl.12953

[39] Hogg CJ. Translating genomic advances into biodiversity conservation. Nat Rev Genet. 2024;25(5):362–73. doi: 10.1038/s41576-023-00671-0 38012268

[40] McLaughlin RW, Cochran PA, Dowd SE. Metagenomic analysis of the gut microbiota of the Timber Rattlesnake, *Crotalus horridus*. Mol Biol Rep. 2015;42(7):1187–95. doi: 10.1007/s11033-015-3854-1 25663091

[41] Tian Z, Pu H, Cai D, Luo G, Zhao L, Li K, et al. Characterization of the bacterial microbiota in different gut and oral compartments of splendid japalure (*Japalura sensu lato*). BMC Vet Res. 2022;18(1):205. doi: 10.1186/s12917-022-03300-w 35624481 PMC9137078

[42] Price JT, Paladino FV, Lamont MM, Witherington BE, Bates ST, Soule T. Characterization of the juvenile green turtle (*Chelonia mydas*) microbiome throughout an ontogenetic shift from pelagic to neritic habitats. PLoS One. 2017;12(5):e0177642. doi: 10.1371/journal.pone.0177642 28493980 PMC5426784

[43] Costello EK, Gordon JI, Secor SM, Knight R. Postprandial remodeling of the gut microbiota in Burmese pythons. ISME J. 2010;4(11):1375–85. doi: 10.1038/ismej.2010.71 20520652 PMC3923499

[44] Lupescu I, Baraitareanu S. Emerging diseases associated with “new companion animals”: review in zoonoses transmitted by reptiles. Sci Works Ser C Vet Med. 2015;61:135–8.

[45] Lee SW, Wendy W. Antibiotic and heavy metal resistance of *Aeromonas hydrophila* and *Edwardsiella tarda* isolated from red hybrid tilapia (*Oreochromis* spp.) coinfected with motile aeromonas septicemia and edwardsiellosis. Vet World. 2017;10(7):803–7. doi: 10.14202/vetworld.2017.803-807 28831226 PMC5553151

[46] Janda JM, Abbott SL. The genus *Aeromonas*: taxonomy, pathogenicity, and infection. Clin Microbiol Rev. 2010;23(1):35–73. doi: 10.1128/CMR.00039-09 20065325 PMC2806660

[47] Semwal A, Kumar A, Kumar N. A review on pathogenicity of *Aeromonas hydrophila* and their mitigation through medicinal herbs in aquaculture. Heliyon. 2023;9(3):e14088. doi: 10.1016/j.heliyon.2023.e14088 36938468 PMC10018484

[48] Abd El-Ghany WA. A review on aeromoniasis in poultry: a bacterial disease of zoonotic nature. J Infect Dev Ctries. 2023;17(1):1–9. doi: 10.3855/jidc.17186 36795920

[49] Hirai Y, Asahata-Tago S, Ainoda Y, Fujita T, Kikuchi K. Edwardsiella tarda bacteremia. A rare but fatal water- and foodborne infection: Review of the literature and clinical cases from a single centre. Can J Infect Dis Med Microbiol. 2015;26(6):313–8. doi: 10.1155/2015/702615 26744588 PMC4692300

[50] Hasegawa K, Kenya M, Suzuki K, Ogawa Y. Characteristics and prognosis of patients with *Edwardsiella tarda* bacteremia at a single institution, Japan, 2005-2022. Ann Clin Microbiol Antimicrob. 2022;21(1):56. doi: 10.1186/s12941-022-00548-w 36476326 PMC9730647

[51] Vitovec J, Aldova E, Vladik P, Krovacek K. Enteropathogenicity of *Plesiomonas shigelloides* and *Aeromonas* spp. in experimental mono- and coinfection with *Cryptosporidium parvum* in the intestine of neonatal BALB/c mice. Comp Immunol Microbiol Infect Dis. 2001;24(1):39–55. doi: 10.1016/s0147-9571(00)00012-6 11131040

[52] Mendoza-Roldan JA, Mendoza-Roldan MA, Otranto D. Reptile vector-borne diseases of zoonotic concern. Int J Parasitol Parasites Wildl. 2021;15:132–42. doi: 10.1016/j.ijppaw.2021.04.007 34026483 PMC8121771

[53] Tang S, Li Y, Huang C, Yan S, Li Y, Chen Z, et al. Comparison of gut microbiota diversity between captive and wild Tokay Gecko (Gekko gecko). Front Microbiol. 2022;13:897923. doi: 10.3389/fmicb.2022.897923 35783386 PMC9248866

[54] Rhimi W, Mendoza-Roldan J, Aneke CI, Mosca A, Otranto D, Alastruey-Izquierdo A, et al. Role of lizards as reservoirs of pathogenic yeasts of zoonotic concern. Acta Trop. 2022;231:106472. doi: 10.1016/j.actatropica.2022.106472 35443196

[55] Ugochukwu ICI, Mendoza-Roldan JA, Rhimi W, Miglianti M, Odigie AE, Mosca A, et al. Snakes as sentinel of zoonotic yeasts and bio-indicators of environmental quality. Sci Rep. 2024;14(1):22491. doi: 10.1038/s41598-024-73195-0 39341972 PMC11438876

[56] Ugochukwu ICI, Mendoza-Roldan JA, Miglianti M, Palazzo N, Odigie AE, Otranto D, et al. Virulence profile of pathogenic yeasts from snakes: alternative ways for antifungal strategies. PLoS One. 2025;20(3):e0318703. doi: 10.1371/journal.pone.0318703 40072936 PMC11902152

[57] Rhimi W, Aneke CI, Annoscia G, Camarda A, Mosca A, Cantacessi C, et al. Virulence and in vitro antifungal susceptibility of *Candida albicans* and *Candida catenulata* from laying hens. Int Microbiol. 2021;24(1):57–63. doi: 10.1007/s10123-020-00141-1 32772220 PMC7873078

[58] Bogan JE, Mason AK, Mishel K, Garner MM, Walden HDS, Childress A, et al. Comparison of sampling techniques and diagnostic tests for *Cryptosporidium serpentis* in eastern indigo snakes (*Drymarchon couperi*). Am J Vet Res. 2024;85(10):ajvr.24.05.0136. doi: 10.2460/ajvr.24.05.0136 39116915

[59] Hawthorne WH, Bogan JE, Ball R, Goessling JM. Physiological responses of eastern indigo snakes (*Drymarchon couperi*) infected with *Cryptosporidium serpentis*. J Herpetol Med Surg. 2024;34:137–44. doi: 10.5818/jhms-d-22-00016

[60] Colombo AL, Padovan AC, Chaves GM. Current knowledge of *Trichosporon* spp. and Trichosporonosis. Clin Microbiol Rev. 2011;24:682–700. doi: 10.1128/CMR.00003-11 21976604 PMC3194827

[61] Lo C, Kang CL, Sun PL, Yu PH, Li WT. Disseminated fungal infection and fungemia caused by *Trichosporon asahii* in a captive plumed basilisk (*Basiliscus plumifrons*). J Fungi. 2021;7. doi: 10.3390/jof7121003 PMC8707992 34946986

[62] Paré JA, Conley KJ. Mycotic diseases of reptiles. In: Jacobson ER, Garner MM, editors. Infectious diseases and pathology of reptiles. 2nd ed. CRC Press; 2021. pp. 795–857.

[63] Allender MC, Raudabaugh DB, Gleason FH, Miller AN. The natural history, ecology, and epidemiology of *Ophidiomyces ophiodiicola* and its potential impact on free-ranging snake populations. Fungal Ecology. 2015;17:187–96.

[64] Lorch JM, Knowles S, Lankton JS, Michell K, Edwards JL, Kapfer JM, et al. Snake fungal disease: an emerging threat to wild snakes. Philos Trans R Soc Lond B Biol Sci. 2016;371(1709):20150457. doi: 10.1098/rstb.2015.0457 28080983 PMC5095536

[65] Romer AS, Grisnik M, Dallas JW, Sutton W, Murray CM, Hardman RH, et al. Effects of snake fungal disease (ophidiomycosis) on the skin microbiome across two major experimental scales. Conserv Biol. 2025;39(2):e14411. doi: 10.1111/cobi.14411 39530499 PMC11959348

[66] Meyer J, Loncaric I, Richter B, Spergser J. Fatal *Purpureocillium lilacinum* pneumonia in a green tree python. J Vet Diagn Invest. 2018;30(2):305–9. doi: 10.1177/1040638717750430 29271312 PMC6505865

[67] Nawange SR, Singh K, Naidu J, Singh SM. Naturally acquired systemic dual infection caused by *Candida famata* (*Debaryomyces hansenii*) and *Candida catenulata* in albino rats bred for sale in the market at Jabalpur (Madhya Pradesh), India. Mycoses. 2010;53(2):173–5. doi: 10.1111/j.1439-0507.2008.01686.x 19298355

[68] Buela L, Cuenca M, Sarmiento J, Peláez D, Mendoza AY, Cabrera EJ, et al. Role of Guinea Pigs (*Cavia porcellus*) raised as livestock in Ecuadorian Andes as Reservoirs of Zoonotic Yeasts. Animals (Basel). 2022;12(24):3449. doi: 10.3390/ani12243449 36552369 PMC9774381

[69] Paiva PRSO, Grego KF, Lima VMF, Nakamura AA, da Silva DC, Meireles MV. Clinical, serological, and parasitological analysis of snakes naturally infected with *Cryptosporidium serpentis*. Vet Parasitol. 2013;198(1–2):54–61. doi: 10.1016/j.vetpar.2013.08.016 24041484

[70] O’Hanlon BM, Bogan JE, Godwin JC, Hoffman M, Smith LL, Chandler HC, et al. *Cryptosporidium serpentis* surveillance in free-ranging snakes to inform a reintroduction strategy for the Eastern Indigo Snake (*Drymarchon couperi*). J Wildl Dis. 2023;59(1):176–80. doi: 10.7589/JWD-D-22-00055 36584345

[71] Cerveny SNS, Garner MM, D’Agostino JJ, Sekscienski SR, Payton ME, Davis MR. Evaluation of gastroscopic biopsy for diagnosis of *Cryptosporidium* sp. infection in snakes. J Zoo Wildl Med. 2012;43(4):864–71. doi: 10.1638/2012-0143.1 23272355

[72] Guo Y, Cebelinski E, Matusevich C, Alderisio KA, Lebbad M, McEvoy J. Subtyping novel zoonotic pathogen *Cryptosporidium* chipmunk genotype I. J Clin Microbiol. 2015;53:1648–54. doi: 10.1128/jcm.03436-14 25762767 PMC4400750

[73] Bujila I, Troell K, Fischerström K, Nordahl M, Killander G, Hansen A, et al. *Cryptosporidium* chipmunk genotype I - an emerging cause of human cryptosporidiosis in Sweden. Infect Genet Evol. 2021;92:104895. doi: 10.1016/j.meegid.2021.104895 33971308

[74] Chalmers RM, Smith R, Elwin K, Clifton-Hadley FA, Giles M. Epidemiology of anthroponotic and zoonotic human cryptosporidiosis in England and Wales, 2004–2006. Epidemiol Infect. 2011;139:700–12. doi: 10.1017/s0950268810001688 20619076

[75] Ryan U, Zahedi A, Feng Y, Xiao L. An update on zoonotic *Cryptosporidium* species and genotypes in humans. Animals. 2021. doi: 10.3390/ani11113307 PMC8614385 34828043

[76] Nguyen KB, Duncan LW. Steinernema diaprepesi n. sp. (Rhabditida: Steinernematidae), a parasite of the citrus root weevil *Diaprepes abbreviatus* (L) (Coleoptera: Curculionidae). J Nematol. 2002;34(2):159–70. 19265926 PMC2620540

