# Supplementary material for: Cloacal microbiome variation in wild and captive Eastern Indigo Snakes (Drymarchon couperi) with and without Cryptosporidium serpentis infection
Source: PLoS One. 2026 Jul 9;21(7):e0350824. doi: 10.1371/journal.pone.0350824 (PMC13349102; doi:10.1371/journal.pone.0350824)
Supplement: S1 Fig — (A) A boxplot is shown comparing the Shannon diversity between wild South Florida snakes (SFL) and headstarted snakes from a private reserve (ABRP). The p-value from the pairwise t-test is shown above the boxplot. (B) A PCoA plot is shown comparing the beta-diversity between wild South Florida snakes (SFL) and headstarted snakes from a private reserve (ABRP). The F-score and p-value are shown above the plot. (DOCX) [file pone.0350824.s001.docx]

**
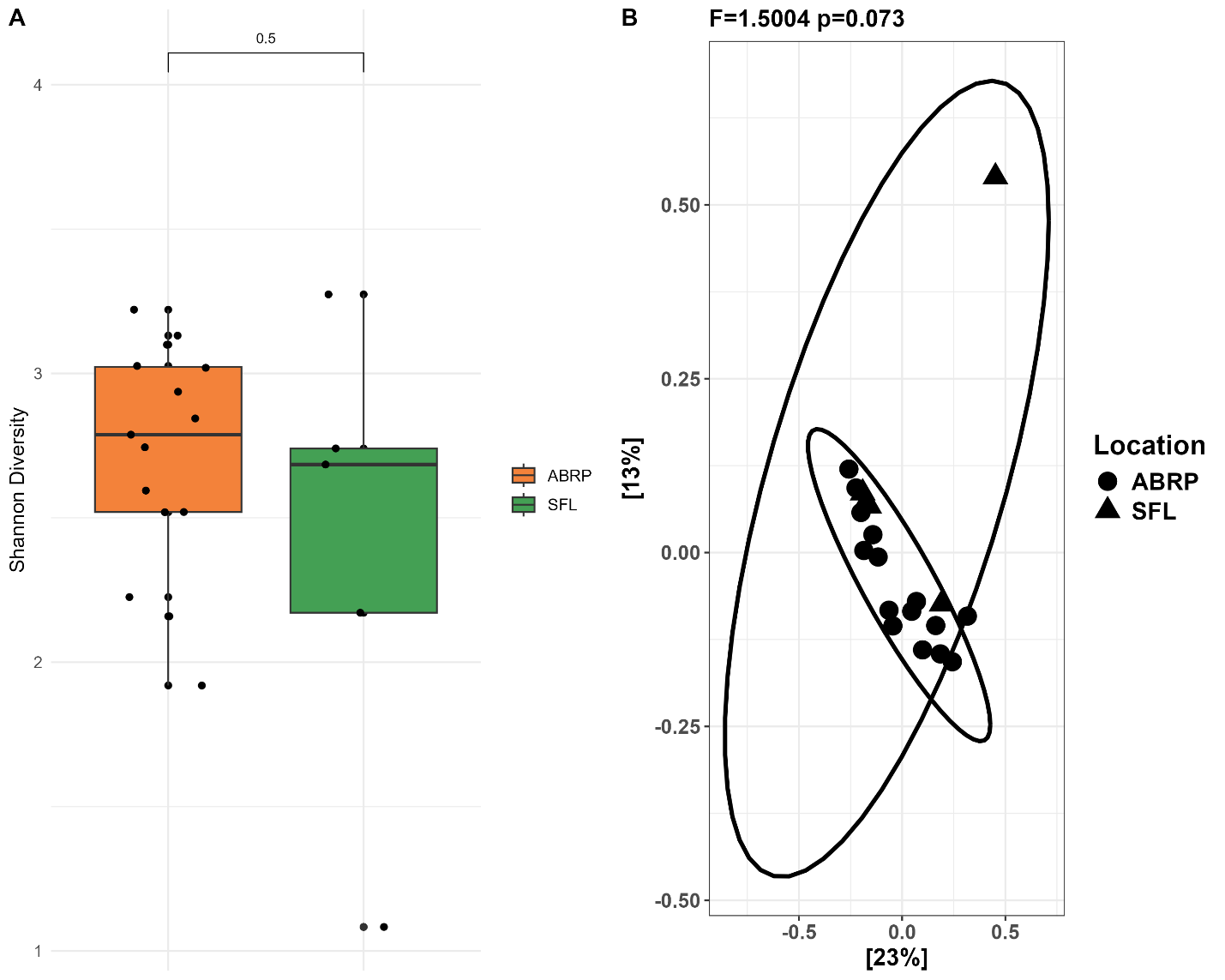
Supplemental Figure S1: There is no significant differences in Shannon diversity or Bray-Curtis beta diversity between wild and headstarted snakes.**

(**A**) A boxplot is shown comparing the Shannon diversity between wild South Florida snakes (SFL) and headstarted snakes from a private reserve (ABRP). The p-value from the pairwise t-test is shown above the boxplot. (**B**) A PCoA plot is shown comparing the beta-diversity between wild South Florida snakes (SFL) and headstarted snakes from a private reserve (ABRP). The F-score and p-value are shown above the plot.
