# Supplementary material for: Cloacal microbiome variation in wild and captive Eastern Indigo Snakes (Drymarchon couperi) with and without Cryptosporidium serpentis infection
Source: PLoS One. 2026 Jul 9;21(7):e0350824. doi: 10.1371/journal.pone.0350824 (PMC13349102; doi:10.1371/journal.pone.0350824)
Supplement: S2 Fig — A bar plot is shown indicating the abundance-weighted fraction of k-mer signatures from each sample that are classified to each database. The y-axis represents the fraction of k-mers from the sample that align to each database. The gray portions of the bars depict proportions of the samples that are unclassified. (DOCX) [file pone.0350824.s002.docx]

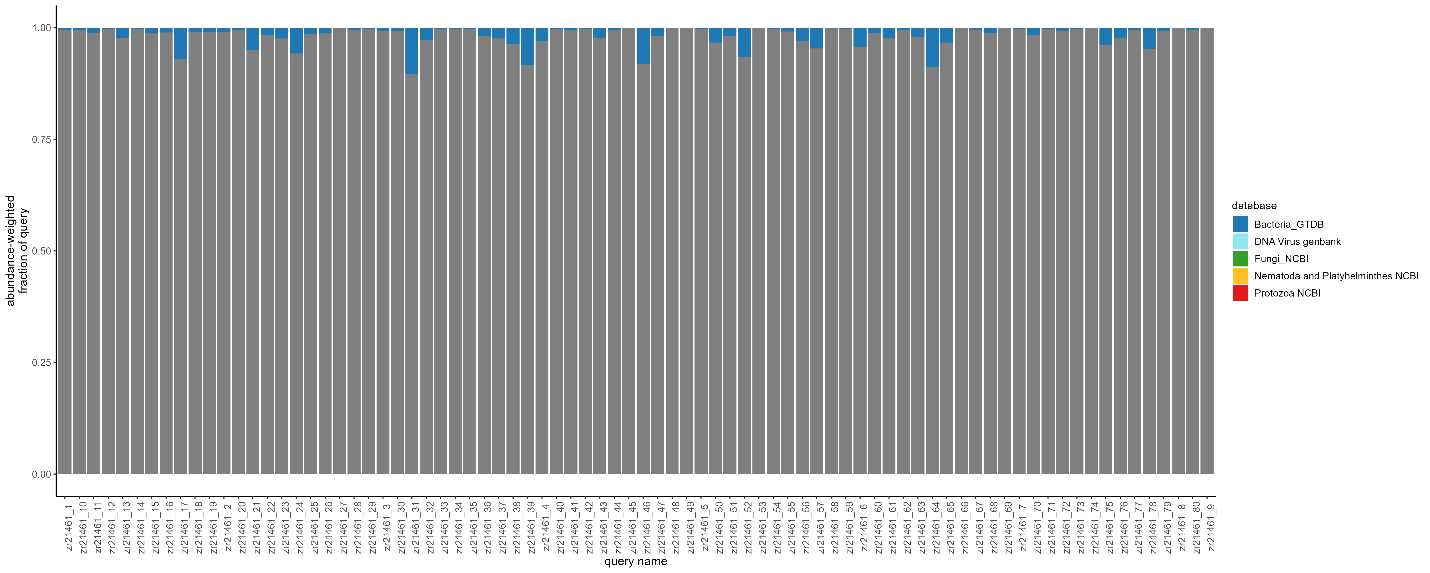


**Supplemental Figure S2: The cloacal microbiome of EIS is largely unclassified.** A bar plot is shown indicating the abundance-weighted fraction of k-mer signatures from each sample that are classified to each database. The y-axis represents the fraction of k-mers from the sample that align to each database. The gray portions of the bars depict proportions of the samples that are unclassified.
