# Supplementary material for: Cloacal microbiome variation in wild and captive Eastern Indigo Snakes (Drymarchon couperi) with and without Cryptosporidium serpentis infection
Source: PLoS One. 2026 Jul 9;21(7):e0350824. doi: 10.1371/journal.pone.0350824 (PMC13349102; doi:10.1371/journal.pone.0350824)
Supplement: S3 Fig — A bar plot is shown indicating the abundance-weighted fraction of k-mer signatures from each sample that are classified to the kingdoms Bacteria, Fungi, Virus, Protozoa, and Metazoa. The y-axis represents the fraction of k-mers from the sample that align to each kingdom. (DOCX) [file pone.0350824.s003.docx]

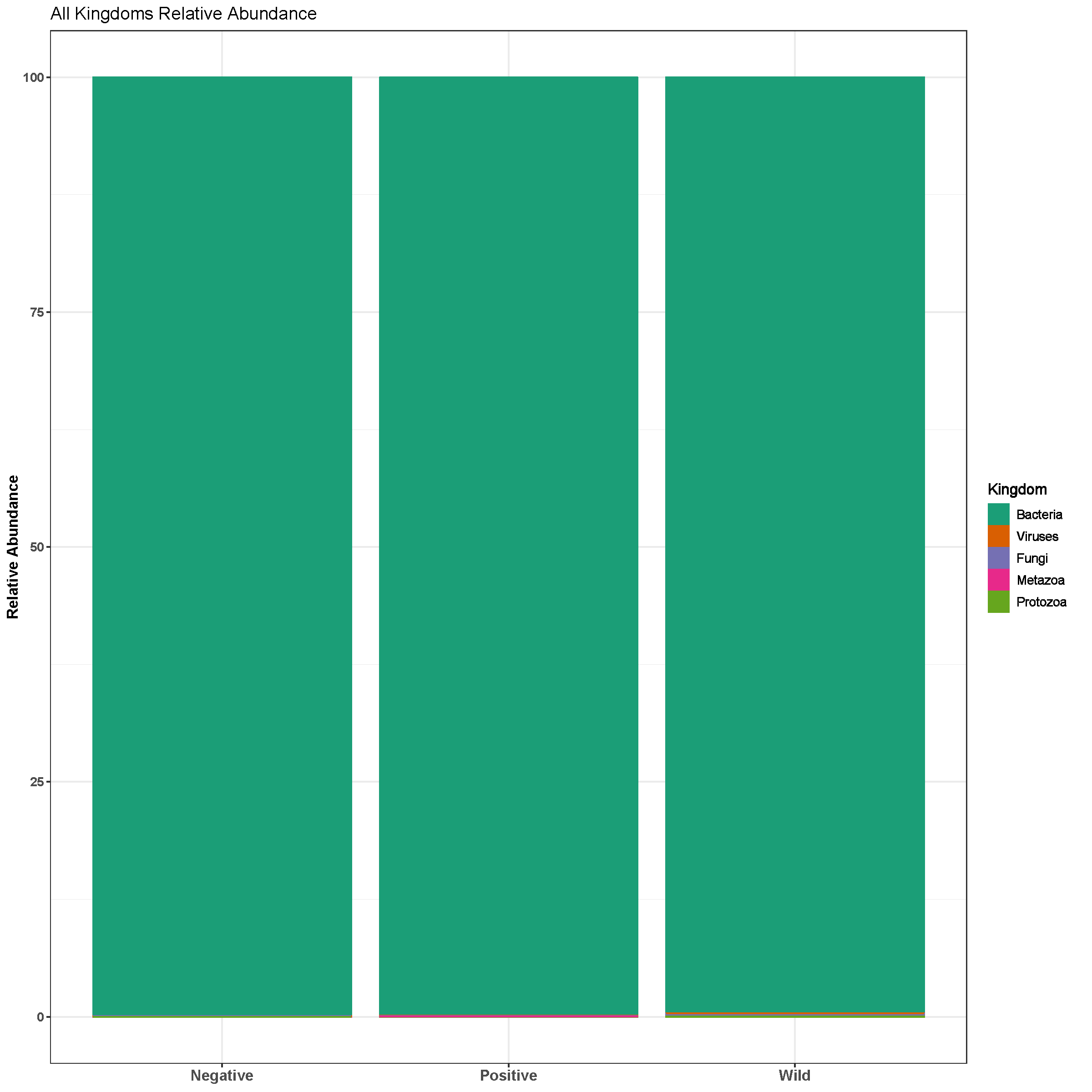


**Supplemental Figure S3: The classified cloacal microbiome of EIS is dominated by bacteria.** A baplot is shown indicating the abundance-weighted fraction of k-mer signatures from each sample that are classified to the kingdoms Bacteria, Fungi, Virus, Protozoa, and Metazoa. The y-axis represents the fraction of k-mers from the sample that align to each kingdom.
