# Supplementary material for: Cloacal microbiome variation in wild and captive Eastern Indigo Snakes (Drymarchon couperi) with and without Cryptosporidium serpentis infection
Source: PLoS One. 2026 Jul 9;21(7):e0350824. doi: 10.1371/journal.pone.0350824 (PMC13349102; doi:10.1371/journal.pone.0350824)
Supplement: S4 Fig — Two linear regression plots are shown indicating the Shannon Diversity of the classified cloacal microbiomes compared with (A) snake age in days and (B) temperature in Farenheit when the sample was collected. P-values are labeled in each plot. Sample points in the figures are shaped by snake sex. (DOCX) [file pone.0350824.s004.docx]

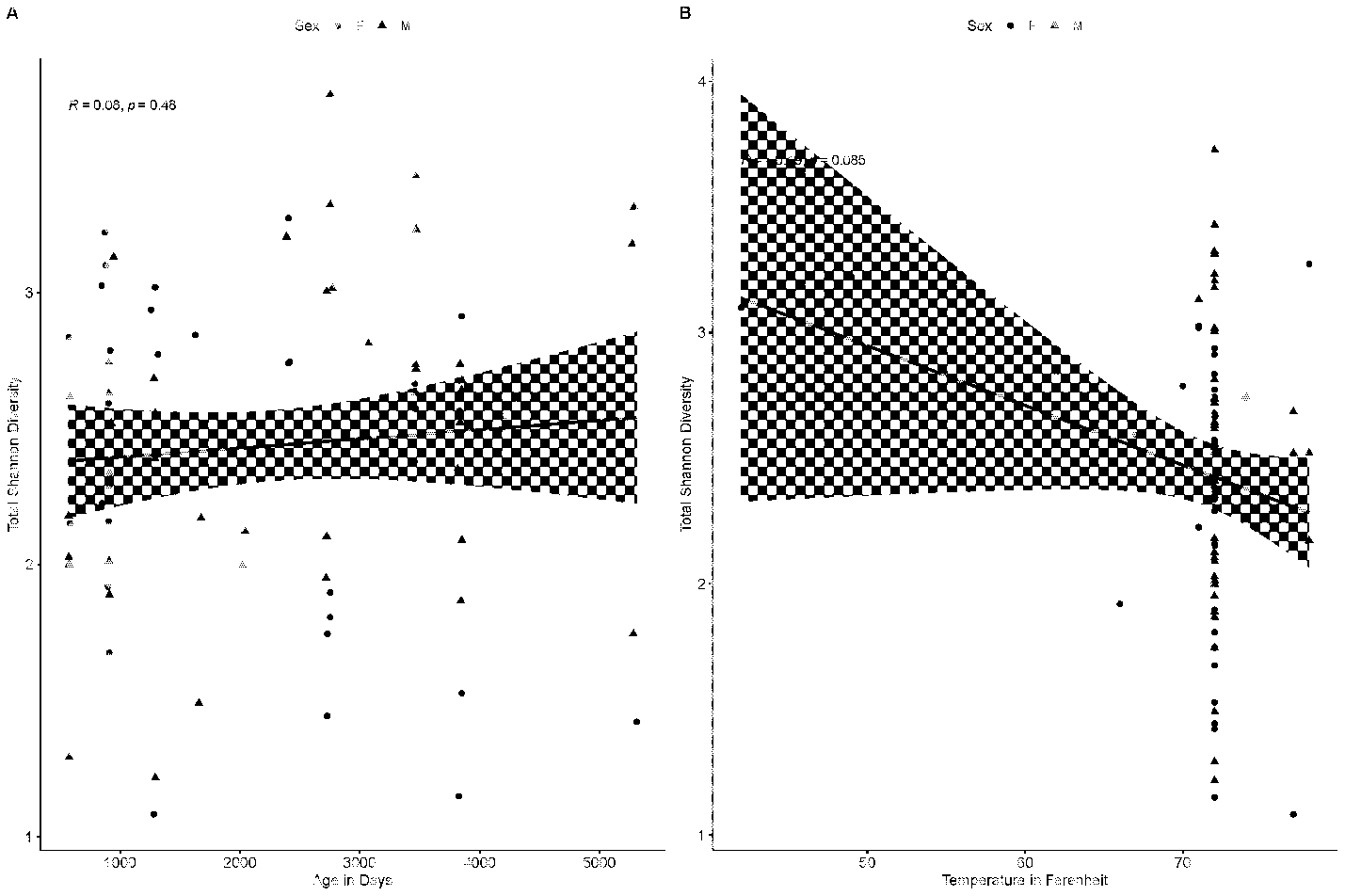


**Supplemental Figure S4: The classified cloacal microbiome richness is not correlated with temperate, age, or sex of the snake.** Two linear regression plots are shown indicating the Shannon Diversity of the classified cloacal microbiomes compared with (**A**) snake Age in Days and (**B**) temperature when the sample was collected. P-values are labeled in each plot. Sample points in the figures are shaped by snake sex.
