# Supplementary material for: Cloacal microbiome variation in wild and captive Eastern Indigo Snakes (Drymarchon couperi) with and without Cryptosporidium serpentis infection
Source: PLoS One. 2026 Jul 9;21(7):e0350824. doi: 10.1371/journal.pone.0350824 (PMC13349102; doi:10.1371/journal.pone.0350824)
Supplement: S5 Fig — (Top) A Venn diagram is shown depicting the number of unique protozoan species observed between the three groups of snakes in this study. The table below the diagram shows the kingdom and species of the four protozoan taxa and the groups of snakes they were observed in. (DOCX) [file pone.0350824.s005.docx]

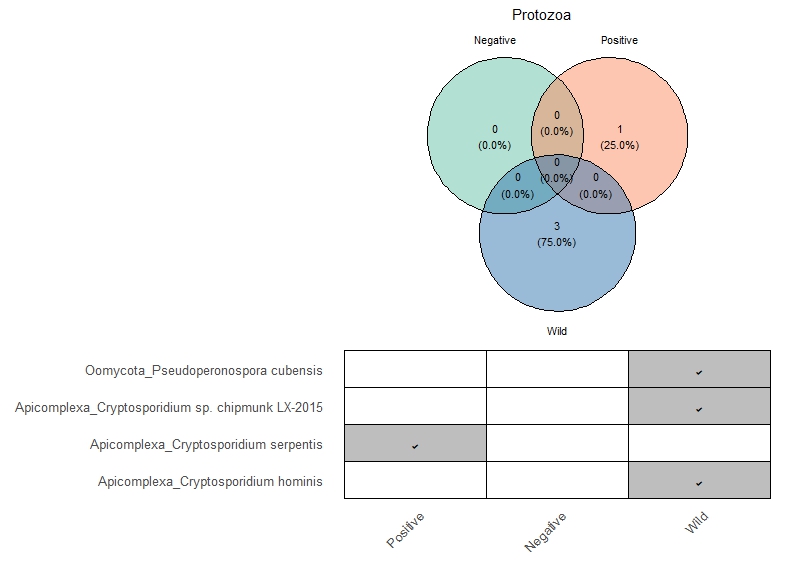


**Supplemental Figure S5: *Cryptosporidium serpentis* is the only protist observed in captive snakes while the wild snakes contain more eukaryotic diversity.** (Top) A Venn diagram is shown depicting the number of unique protozoan species observed between the three groups of snakes in this study. The table below the diagram shows the kingdom and species of the four protozoan taxa and the groups of snakes they were observed in.
