# Supplementary material for: Cloacal microbiome variation in wild and captive Eastern Indigo Snakes (Drymarchon couperi) with and without Cryptosporidium serpentis infection
Source: PLoS One. 2026 Jul 9;21(7):e0350824. doi: 10.1371/journal.pone.0350824 (PMC13349102; doi:10.1371/journal.pone.0350824)
Supplement: Attachment — Submitted filename: Response to Reviewers.docx [file pone.0350824.s007.docx]

**Reviewer 1**

I would like to congratulate the authors on their work and on the manuscript entitled “Cloacal microbiome variation in wild and captive Eastern Indigo Snakes (Drymarchon couperi) with and without Cryptosporidium serpentis infection”. This is an original study focusing on a relatively understudied aspect of reptile ecology, health, and conservation. The introduction provides a strong background and justifies the relevance of the research. Materials and Methods section adequately describes sampling of snakes, and explains metagenomic and statistical analysis methods in great detail. The discussion summarizes the results without major overstatements and creates a logical link between microbial composition and organismal health. However, there are some minor inconsistencies, which I highlight below. I recommend that the authors to use the up-to-date names of bacterial phyla. While the study is technically sound, I suggest minor revision to address some small imprecisions before publication.

**Our answer:**

We thank the reviewer for their constructive feedback. We have revised the manuscript accordingly and made the appropriate changes.

**Comment 1:** Recommend using up-to-date names of bacterial phyla.

**Response 1:** Thank you for pointing this out. Updated bacterial phylum taxonomy was incorporated in the Results and Discussion sections, replacing outdated names and including Pseudomonadota, Actinomycetota, Bacteroidota, and Bacillota (lines 216-217, lines 320-330). Figures were also updated.

**Reviewer 2**

This manuscript describes a shotgun metagenomic study of the cloacal microbiome in the Eastern Indigo Snake (Drymarchon couperi), comparing wild and captive populations with a focus on Cryptosporidium serpentis infection. The study is timely, as reptile microbiome data remains sparse compared to other vertebrates, and the move from amplicon sequencing to a shotgun approach is a significant methodological step forward for this field.

While the study design is sound and the results are compelling, the manuscript would benefit from tightening up in several areas. The Introduction is a bit repetitive regarding the factors influencing reptile microbiomes (diet, geography, etc.) these sections should be condensed to keep the focus on the Eastern Indigo Snake and the study's specific objectives, which should be stated more explicitly at the end of the section.

In the Materials and Methods, there are some inconsistencies in the reported sample sizes for wild snakes across different paragraphs. This needs to be corrected for clarity. Additionally, please specify which reference genome was used for host read filtering, as this is critical for reproducibility.

The Results clearly highlight the higher microbial richness in wild individuals and identify taxa potentially linked to C. serpentis infection. The low detection rate of the parasite itself, despite confirmed infection, is a fair observation, and the discussion of intermittent shedding is a plausible explanation. However, the Discussion could be more concise, particularly in the sections covering bacterial and fungal associations.

A key limitation that should be more formally acknowledged is the lack of direct environmental sampling (soil, water, enclosure surfaces). Without this, the role of environmental exposure remains an inference. Furthermore, the high proportion of unclassified reads while common in non-model wildlife studies warrants a brief mention regarding the current limitations of taxonomic databases for reptiles.

The figures are generally informative, though the labeling and legends could be cleaner to improve readability. Overall, this is a well-executed study that provides valuable baseline data for reptile conservation and disease ecology.

Recommendation: Minor Revision.

**Our answer:**

We thank the reviewer for their constructive and helpful feedback. We have revised the manuscript accordingly and made the appropriate changes for publication.

**Comment 1:** Introduction is repetitive regarding the factors influencing reptile microbiomes (diet, geography, etc.) and objectives should be clearer.

**Response 1:** We appreciate this suggestion and have revised the introduction to reduce redundancy. Edits can be seen in the “Track Changes” document. We have also added a clear objective and hypothesis (lines 92-99).

**Comment 2:** Inconsistency in sample size reporting.

**Response 2:** The sample size was corrected to include 60 captive snakes (30 infected, 30 non-infected) and 20 wild animals (lines 181-182).

**Comment 3:** Specify reference genome used for host read filtering.

**Response 3:** Thank you for the comment. Reads that survived quality trimming were mapped against the genome of *Drymarchon corais* ([GCA_043091225.1](https://nam11.safelinks.protection.outlook.com/?url=https%3A%2F%2Fwww.ncbi.nlm.nih.gov%2Fdatasets%2Fgenome%2FGCA_043091225.1%2F&data=05%7C02%7Ccbrown77%40tulane.edu%7C15a8a5a14db34e93656008de8aa1ba58%7C9de9818325d94b139fc34de5489c1f3b%7C0%7C0%7C639100624421129686%7CUnknown%7CTWFpbGZsb3d8eyJFbXB0eU1hcGkiOnRydWUsIlYiOiIwLjAuMDAwMCIsIlAiOiJXaW4zMiIsIkFOIjoiTWFpbCIsIldUIjoyfQ%3D%3D%7C0%7C%7C%7C&sdata=73O0sU62jC1JfdKt6LKaDctgaEKGzIzO%2BBjhBoifJzw%3D&reserved=0)) using Burrows-Wheeler Alignment (BWA) v.0.7.18 (lines 164-165).

**Comment 4:** Bacterial and fungal discussion sections could be trimmed down.

**Response 4:** The bacterial and fungal discussion sections were trimmed and made more concise. This is included in the new manuscript (now lines 318-408) and the edits made can be visually seen in the “Track Changes” document.

**Comment 5:** Lack of environmental sampling should be acknowledged.

**Response 5:** We agree and have expanded the limitations section to include this in the new manuscript (lines 355-360 & 466-471).

**Comment 6:** High proportion of unclassified reads should be discussed.

**Response 6:** We have added a statement addressing the large proportion of unclassified reads and mentioned the limitations in current taxonomic databases for non-model reptile species (lines 472-485).

**Comment 7:** Figures and legends could be improved.

**Response 7:** Figure 1 has been updated to reflect the current taxonomy names of the bacterial phyla detected in this study. All Figure legends have been updated in greater detail to better guide the reader in interpreting the results.
