# Supplementary material for: Cloacal microbiome variation in wild and captive Eastern Indigo Snakes (Drymarchon couperi) with and without Cryptosporidium serpentis infection
Source: PLoS One. 2026 Jul 9;21(7):e0350824. doi: 10.1371/journal.pone.0350824 (PMC13349102; doi:10.1371/journal.pone.0350824)
Supplement: Attachment — Submitted filename: Response_to_Reviewers_auresp_2.docx [file pone.0350824.s008.docx]

**Reviewer 1**

I commend the authors for addressing my suggestions. However, there are still some minor issues that should be accounted for. I recommend a minor revision for now.

**Our answer:**

We thank the reviewer for their constructive feedback. We have revised the manuscript accordingly and made the appropriate changes.

**Comment 1:** I recommend that the p-value is reported with a lowercase italicized “p” throughout the text.

**Response 1:** Thank you for pointing this out. All p-values were changed and italicized in both the text and figures (please see updated manuscript and track changes document).

**Comment 2:** Lines 158-159: Since most individuals in the “wild” group were mostly rewilded rather than truly wild-hatched, the authors should provide a clearer justification for grouping them as “wild” as this may influence microbiome composition. Were all individuals collected in the private reserve previously reintroduced? Could they differ from truly wild individuals since hatching? I think a comparison between truly wild and headstarted individuals would be justified.

**Response 2:** Great point. All individuals in our study sampled at the private reserve were headstarted. We have run another analysis indicating there was no significant difference in community composition between headstarted individuals and true wild individuals (lines 218-220 in track changes document). Please also see attached supplemental figure S1 comparing headstarted individuals to truly wild individuals (lines 519-525 in track changes document). Furthermore, extrapolating from two previous lizard studies (Eliades et al. 2021 & Forehand et. al. 2025), cloacal microbiomes were indistinguishable between headstarted individuals and true wild individuals after 2 months post-release (References 11 and 19) which supports our findings too.

**Comment 3:** Diversity is not the same thing as richness. The Shannon diversity index incorporates both species richness and evenness, but it is not a direct measure of richness alone. “richness” → “diversity”

**Response 3:** Thank you for clarifying this. Changes were made (see lines 195, 222-225 & 376-377, 537) in track changes document).

**Comment 4:** Line 234: On Fig. 1A diversity is displayed, not richness Instead of “significantly richer,” I suggest “significantly more diverse.”

**Response 4:** Changed to, “wild snakes exhibited significantly greater Shannon diversity” (see line 224 in track changes document).

**Comment 5:** Lines 241-242: I think virus names should be italicized.

**Response 5:** All virus names are now italicized (lines 231-232 in track changes document).

**Comment 6:** Lines 258-259: Fig.1, The phrasing of the title seems a little overcomplicated. I recommend revising the sentence to: “Diverse microbial taxa from multiple kingdoms identified in cloacal samples,” or something similar.

**Response 6:** Title changed to: “Fig 1. Diverse microbial taxa from multiple kingdoms are observed in the cloacal samples” (Lines 248-249).

**Comment 7:** Lines 271-272: Be aware that these taxa are not all kingdoms, and are not at the same taxonomic level, so I recommend rewording the sentence.

**Response 7:** We changed to “across five major viral and microbial taxonomic groups”. (Lines 255-256 in track changes document).

**Comment 8:** Line 282: In my opinion, it is better to write it as “p<0.001.”

**Response 8:** Changed to P<0.001 (Line 262).

**Comment 9:** Lines 330-331: I recommend rephrasing the sentence, to avoid any overstatements. See Hernández et al., 2025.

**Response 9:** Changed from “reptilian cloacal microbiome” to “ophidian cloacal microbiome” (line 310 in track changes document).

**Comment 10:** Lines 338–339: “…that can influence overall organism health” — I think a few words about the link between microbial diversity and organismal health would strengthen the results.

**Response 10:** We agree. I’ve added this, “We have demonstrated that shotgun metagenomic sequencing provides valuable insight into the cloacal microbiome. This approach may also aid in identifying microbial associations relevant to disease status and overall snake health, as shifts in microbial diversity and community composition may reflect dysbiosis and alterations in immune function.” (Lines 318-321 in track changes document).

**Comment 11:** Line 353: “phylum” instead of “phyla.”

**Response 11:** Corrected (line 343 in track changes document).

**Comment 12:** Line 619: The references should be revised so that Latin names are italicized.

**Response 12:** All references are now revised so that Latin names are italicized (lines 558-783 in track changes document).

**Comment 13:** Figure 1: Species names should be italicized.

**Response 13:** Species names are corrected and now italicized (see Figure 1).

**Comment 14:** Figure 2: There are dots in the legend, and it is not italicized. Also, the “p” should be lowercase.

**Response 14:** Corrected (see Figure 2).
