# Supplementary material for: Cloacal microbiome variation in wild and captive Eastern Indigo Snakes (Drymarchon couperi) with and without Cryptosporidium serpentis infection
Source: PLoS One. 2026 Jul 9;21(7):e0350824. doi: 10.1371/journal.pone.0350824 (PMC13349102; doi:10.1371/journal.pone.0350824)
Supplement: Peer review history

# Peer review: Decision Letter 0

**DOI:** 10.1371/journal.pone.0350824.r001

**Authors:** Edvard Mizsei

15 Mar 2026

PONE-D-26-07884

Cloacal microbiome variation in wild and captive Eastern Indigo Snakes (Drymarchon couperi) with and without Cryptosporidium serpentis infection

PLOS One

Dear Dr. Brown,

Thank you for submitting your manuscript to PLOS ONE. After careful consideration, we feel that it has merit but does not fully meet PLOS ONE’s publication criteria as it currently stands. Therefore, we invite you to submit a revised version of the manuscript that addresses the points raised during the review process.

Please submit your revised manuscript by Apr 29 2026 11:59PM. If you will need more time than this to complete your revisions, please reply to this message or contact the journal office at plosone@plos.org. When you're ready to submit your revision, log on to https://www.editorialmanager.com/pone/ and select the 'Submissions Needing Revision' folder to locate your manuscript file.

Please include the following items when submitting your revised manuscript:

- A letter that responds to each point raised by the academic editor and reviewer(s). You should upload this letter as a separate file labeled 'Response to Reviewers'.
- A marked-up copy of your manuscript that highlights changes made to the original version. You should upload this as a separate file labeled 'Revised Manuscript with Track Changes'.
- An unmarked version of your revised paper without tracked changes. You should upload this as a separate file labeled 'Manuscript'.

-->If you would like to make changes to your financial disclosure, please include your updated statement in your cover letter. Guidelines for resubmitting your figure files are available below the reviewer comments at the end of this letter.

If applicable, we recommend that you deposit your laboratory protocols in protocols.io to enhance the reproducibility of your results. Protocols.io assigns your protocol its own identifier (DOI) so that it can be cited independently in the future. For instructions see: https://journals.plos.org/plosone/s/submission-guidelines#loc-laboratory-protocols. Additionally, PLOS ONE offers an option for publishing peer-reviewed Lab Protocol articles, which describe protocols hosted on protocols.io. Read more information on sharing protocols at https://plos.org/protocols?utm_medium=editorial-email&utm_source=authorletters&utm_campaign=protocols.

We look forward to receiving your revised manuscript.

Kind regards,

Edvard Mizsei

Academic Editor

PLOS One

Journal Requirements:

When submitting your revision, we need you to address these additional requirements.

1. Please ensure that your manuscript meets PLOS ONE's style requirements, including those for file naming. The PLOS ONE style templates can be found at

https://journals.plos.org/plosone/s/file?id=wjVg/PLOSOne_formatting_sample_main_body.pdf and

https://journals.plos.org/plosone/s/file?id=ba62/PLOSOne_formatting_sample_title_authors_affiliations.pdf

2.  To comply with PLOS One submissions requirements, in your Methods section, please provide additional information regarding the experiments involving animals and ensure you have included details on (1) methods of sacrifice, (2) methods of anesthesia and/or analgesia, and (3) efforts to alleviate suffering.

3. Please expand the acronym “ACLAM” (as indicated in your financial disclosure) so that it states the name of your funders in full.

This information should be included in your cover letter; we will change the online submission form on your behalf.

4. Please include captions for your Supporting Information files at the end of your manuscript, and update any in-text citations to match accordingly. Please see our Supporting Information guidelines for more information: http://journals.plos.org/plosone/s/supporting-information.

5. If the reviewer comments include a recommendation to cite specific previously published works, please review and evaluate these publications to determine whether they are relevant and should be cited. There is no requirement to cite these works unless the editor has indicated otherwise.

6. Please review your reference list to ensure that it is complete and correct. If you have cited papers that have been retracted, please include the rationale for doing so in the manuscript text, or remove these references and replace them with relevant current references. Any changes to the reference list should be mentioned in the rebuttal letter that accompanies your revised manuscript. If you need to cite a retracted article, indicate the article’s retracted status in the References list and also include a citation and full reference for the retraction notice.

[Note: HTML markup is below. Please do not edit.]

Reviewers' comments:

Reviewer's Responses to Questions

**Comments to the Author**

1. Is the manuscript technically sound, and do the data support the conclusions?

The manuscript must describe a technically sound piece of scientific research with data that supports the conclusions. Experiments must have been conducted rigorously, with appropriate controls, replication, and sample sizes. The conclusions must be drawn appropriately based on the data presented.

Reviewer #1: Yes

Reviewer #2: Yes

**********

2. Has the statistical analysis been performed appropriately and rigorously?

Reviewer #1: Yes

Reviewer #2: Yes

**********

3. Have the authors made all data underlying the findings in their manuscript fully available?

The PLOS Data policy requires authors to make all data underlying the findings described in their manuscript fully available without restriction, with rare exception (please refer to the Data Availability Statement in the manuscript PDF file). The data should be provided as part of the manuscript or its supporting information, or deposited to a public repository. For example, in addition to summary statistics, the data points behind means, medians and variance measures should be available. If there are restrictions on publicly sharing data—e.g. participant privacy or use of data from a third party—those must be specified.

Reviewer #1: Yes

Reviewer #2: Yes

**********

4. Is the manuscript presented in an intelligible fashion and written in standard English?

PLOS ONE does not copyedit accepted manuscripts, so the language in submitted articles must be clear, correct, and unambiguous. Any typographical or grammatical errors should be corrected at revision, so please note any specific errors here.

Reviewer #1: Yes

Reviewer #2: Yes

**********

5. Review Comments to the Author

Please use the space provided to explain your answers to the questions above. You may also include additional comments for the author, including concerns about dual publication, research ethics, or publication ethics. (Please upload your review as an attachment if it exceeds 20,000 characters)

Reviewer #1: Review comments to the Authors

I would like to congratulate the authors on their work and on the manuscript entitled “Cloacal microbiome variation in wild and captive Eastern Indigo Snakes (Drymarchon couperi) with and without Cryptosporidium serpentis infection”. This is an original study focusing on a relatively understudied aspect of reptile ecology, health, and conservation. The introduction provides a strong background and justifies the relevance of the research. Materials and Methods section adequately describes sampling of snakes, and explains metagenomic and statistical analysis methods in great detail. The discussion summerizes the results without major overstatements and creates a logical link between microbial composition and organismal health. However, there are some minor incosistencies, which I highlight below. I recommend that the authors to use the up-to-date names of bacterial phyla. While the study is technically sound, I suggest minor revision to address some small imprecisions before publication.

Reviewer #2: This manuscript describes a shotgun metagenomic study of the cloacal microbiome in the Eastern Indigo Snake (Drymarchon couperi), comparing wild and captive populations with a focus on Cryptosporidium serpentis infection. The study is timely, as reptile microbiome data remains sparse compared to other vertebrates, and the move from amplicon sequencing to a shotgun approach is a significant methodological step forward for this field.

While the study design is sound and the results are compelling, the manuscript would benefit from tightening up in several areas. The Introduction is a bit repetitive regarding the factors influencing reptile microbiomes (diet, geography, etc.) these sections should be condensed to keep the focus on the Eastern Indigo Snake and the study's specific objectives, which should be stated more explicitly at the end of the section.

In the Materials and Methods, there are some inconsistencies in the reported sample sizes for wild snakes across different paragraphs. This needs to be corrected for clarity. Additionally, please specify which reference genome was used for host read filtering, as this is critical for reproducibility.

The Results clearly highlight the higher microbial richness in wild individuals and identify taxa potentially linked to C. serpentis infection. The low detection rate of the parasite itself, despite confirmed infection, is a fair observation, and the discussion of intermittent shedding is a plausible explanation. However, the Discussion could be more concise, particularly in the sections covering bacterial and fungal associations.

A key limitation that should be more formally acknowledged is the lack of direct environmental sampling (soil, water, enclosure surfaces). Without this, the role of environmental exposure remains an inference. Furthermore, the high proportion of unclassified reads while common in non-model wildlife studies warrants a brief mention regarding the current limitations of taxonomic databases for reptiles.

The figures are generally informative, though the labeling and legends could be cleaner to improve readability. Overall, this is a well-executed study that provides valuable baseline data for reptile conservation and disease ecology.

Recommendation: Minor Revision.

**********

6. PLOS authors have the option to publish the peer review history of their article (what does this mean?). If published, this will include your full peer review and any attached files.

If you choose “no”, your identity will remain anonymous but your review may still be made public.

**Do you want your identity to be public for this peer review?**  For information about this choice, including consent withdrawal, please see our Privacy Policy.

Reviewer #1: No

Reviewer #2: No

**********

[NOTE: If reviewer comments were submitted as an attachment file, they will be attached to this email and accessible via the submission site. Please log into your account, locate the manuscript record, and check for the action link "View Attachments". If this link does not appear, there are no attachment files.]

To ensure your figures meet our technical requirements, please review our figure guidelines: https://journals.plos.org/plosone/s/figures

You may also use PLOS’s free figure tool, NAAS, to help you prepare publication quality figures: https://journals.plos.org/plosone/s/figures#loc-tools-for-figure-preparation.

NAAS will assess whether your figures meet our technical requirements by comparing each figure against our figure specifications.

# Author comment: Author response to Decision Letter 1

**DOI:** 10.1371/journal.pone.0350824.r002

6 Apr 2026

Reviewer 1

I would like to congratulate the authors on their work and on the manuscript entitled “Cloacal microbiome variation in wild and captive Eastern Indigo Snakes (Drymarchon couperi) with and without Cryptosporidium serpentis infection”. This is an original study focusing on a relatively understudied aspect of reptile ecology, health, and conservation. The introduction provides a strong background and justifies the relevance of the research. Materials and Methods section adequately describes sampling of snakes, and explains metagenomic and statistical analysis methods in great detail. The discussion summarizes the results without major overstatements and creates a logical link between microbial composition and organismal health. However, there are some minor inconsistencies, which I highlight below. I recommend that the authors to use the up-to-date names of bacterial phyla. While the study is technically sound, I suggest minor revision to address some small imprecisions before publication.

Our answer:

We thank the reviewer for their constructive feedback. We have revised the manuscript accordingly and made the appropriate changes.

Comment 1: Recommend using up-to-date names of bacterial phyla.

Response 1: Thank you for pointing this out. Updated bacterial phylum taxonomy was incorporated in the Results and Discussion sections, replacing outdated names and including Pseudomonadota, Actinomycetota, Bacteroidota, and Bacillota (lines 216-217, lines 320-330). Figures were also updated.

Reviewer 2

This manuscript describes a shotgun metagenomic study of the cloacal microbiome in the Eastern Indigo Snake (Drymarchon couperi), comparing wild and captive populations with a focus on Cryptosporidium serpentis infection. The study is timely, as reptile microbiome data remains sparse compared to other vertebrates, and the move from amplicon sequencing to a shotgun approach is a significant methodological step forward for this field.

While the study design is sound and the results are compelling, the manuscript would benefit from tightening up in several areas. The Introduction is a bit repetitive regarding the factors influencing reptile microbiomes (diet, geography, etc.) these sections should be condensed to keep the focus on the Eastern Indigo Snake and the study's specific objectives, which should be stated more explicitly at the end of the section.

In the Materials and Methods, there are some inconsistencies in the reported sample sizes for wild snakes across different paragraphs. This needs to be corrected for clarity. Additionally, please specify which reference genome was used for host read filtering, as this is critical for reproducibility.

The Results clearly highlight the higher microbial richness in wild individuals and identify taxa potentially linked to C. serpentis infection. The low detection rate of the parasite itself, despite confirmed infection, is a fair observation, and the discussion of intermittent shedding is a plausible explanation. However, the Discussion could be more concise, particularly in the sections covering bacterial and fungal associations.

A key limitation that should be more formally acknowledged is the lack of direct environmental sampling (soil, water, enclosure surfaces). Without this, the role of environmental exposure remains an inference. Furthermore, the high proportion of unclassified reads while common in non-model wildlife studies warrants a brief mention regarding the current limitations of taxonomic databases for reptiles.

The figures are generally informative, though the labeling and legends could be cleaner to improve readability. Overall, this is a well-executed study that provides valuable baseline data for reptile conservation and disease ecology.

Recommendation: Minor Revision.

Our answer:

We thank the reviewer for their constructive and helpful feedback. We have revised the manuscript accordingly and made the appropriate changes for publication.

Comment 1: Introduction is repetitive regarding the factors influencing reptile microbiomes (diet, geography, etc.) and objectives should be clearer.

Response 1: We appreciate this suggestion and have revised the introduction to reduce redundancy. Edits can be seen in the “Track Changes” document. We have also added a clear objective and hypothesis (lines 92-99).

Comment 2: Inconsistency in sample size reporting.

Response 2: The sample size was corrected to include 60 captive snakes (30 infected, 30 non-infected) and 20 wild animals (lines 181-182).

Comment 3: Specify reference genome used for host read filtering.

Response 3: Thank you for the comment. Reads that survived quality trimming were mapped against the genome of Drymarchon corais (GCA_043091225.1) using Burrows-Wheeler Alignment (BWA) v.0.7.18 (lines 164-165).

Comment 4: Bacterial and fungal discussion sections could be trimmed down.

Response 4: The bacterial and fungal discussion sections were trimmed and made more concise. This is included in the new manuscript (now lines 318-408) and the edits made can be visually seen in the “Track Changes” document.

Comment 5: Lack of environmental sampling should be acknowledged.

Response 5: We agree and have expanded the limitations section to include this in the new manuscript (lines 355-360 & 466-471).

Comment 6: High proportion of unclassified reads should be discussed.

Response 6: We have added a statement addressing the large proportion of unclassified reads and mentioned the limitations in current taxonomic databases for non-model reptile species (lines 472-485).

Comment 7: Figures and legends could be improved.

Response 7: Figure 1 has been updated to reflect the current taxonomy names of the bacterial phyla detected in this study. All Figure legends have been updated in greater detail to better guide the reader in interpreting the results.

Attachment Submitted filename: Response to Reviewers.docx

# Peer review: Decision Letter 1

**DOI:** 10.1371/journal.pone.0350824.r003

**Authors:** Edvard Mizsei, Edvard Mizsei

8 May 2026

PONE-D-26-07884R1

Cloacal microbiome variation in wild and captive Eastern Indigo Snakes (Drymarchon couperi) with and without Cryptosporidium serpentis infection

PLOS One

Dear Dr. Brown,

Thank you for submitting your manuscript to PLOS ONE. After careful consideration, we feel that it has merit but does not fully meet PLOS ONE’s publication criteria as it currently stands. Therefore, we invite you to submit a revised version of the manuscript that addresses the points raised during the review process.

Please submit your revised manuscript by Jun 22 2026 11:59PM. If you will need more time than this to complete your revisions, please reply to this message or contact the journal office at plosone@plos.org. When you're ready to submit your revision, log on to https://www.editorialmanager.com/pone/ and select the 'Submissions Needing Revision' folder to locate your manuscript file.

Please include the following items when submitting your revised manuscript:

- A letter that responds to each point raised by the academic editor and reviewer(s). You should upload this letter as a separate file labeled 'Response to Reviewers'.
- A marked-up copy of your manuscript that highlights changes made to the original version. You should upload this as a separate file labeled 'Revised Manuscript with Track Changes'.
- An unmarked version of your revised paper without tracked changes. You should upload this as a separate file labeled 'Manuscript'.

If you would like to make changes to your financial disclosure, please include your updated statement in your cover letter. Guidelines for resubmitting your figure files are available below the reviewer comments at the end of this letter.

If applicable, we recommend that you deposit your laboratory protocols in protocols.io to enhance the reproducibility of your results. Protocols.io assigns your protocol its own identifier (DOI) so that it can be cited independently in the future. For instructions see: https://journals.plos.org/plosone/s/submission-guidelines#loc-laboratory-protocols. Additionally, PLOS ONE offers an option for publishing peer-reviewed Lab Protocol articles, which describe protocols hosted on protocols.io. Read more information on sharing protocols at https://plos.org/protocols?utm_medium=editorial-email&utm_source=authorletters&utm_campaign=protocols.

As the corresponding author, your ORCID iD is verified in the submission system and will appear in the published article. PLOS supports the use of ORCID, and we encourage all coauthors to register for an ORCID iD and use it as well. Please encourage your coauthors to verify their ORCID iD within the submission system before final acceptance, as unverified ORCID iDs will not appear in the published article. *Only*  the individual author can complete the verification step; PLOS staff *cannot*  verify ORCID iDs on behalf of authors.

We look forward to receiving your revised manuscript.

Kind regards,

Edvard Mizsei

Academic Editor

PLOS One

Journal Requirements:

1. If the reviewer comments include a recommendation to cite specific previously published works, please review and evaluate these publications to determine whether they are relevant and should be cited. There is no requirement to cite these works unless the editor has indicated otherwise.

2. Please review your reference list to ensure that it is complete and correct. If you have cited papers that have been retracted, please include the rationale for doing so in the manuscript text, or remove these references and replace them with relevant current references. Any changes to the reference list should be mentioned in the rebuttal letter that accompanies your revised manuscript. If you need to cite a retracted article, indicate the article’s retracted status in the References list and also include a citation and full reference for the retraction notice.

[Note: HTML markup is below. Please do not edit.]

Reviewers' comments:

Reviewer's Responses to Questions

**Comments to the Author**

1. If the authors have adequately addressed your comments raised in a previous round of review and you feel that this manuscript is now acceptable for publication, you may indicate that here to bypass the “Comments to the Author” section, enter your conflict of interest statement in the “Confidential to Editor” section, and submit your "Accept" recommendation.

Reviewer #1: (No Response)

**********

2. Is the manuscript technically sound, and do the data support the conclusions?

The manuscript must describe a technically sound piece of scientific research with data that supports the conclusions. Experiments must have been conducted rigorously, with appropriate controls, replication, and sample sizes. The conclusions must be drawn appropriately based on the data presented.

Reviewer #1: Yes

**********

3. Has the statistical analysis been performed appropriately and rigorously?

Reviewer #1: Yes

**********

4. Have the authors made all data underlying the findings in their manuscript fully available?

The PLOS Data policy requires authors to make all data underlying the findings described in their manuscript fully available without restriction, with rare exception (please refer to the Data Availability Statement in the manuscript PDF file). The data should be provided as part of the manuscript or its supporting information, or deposited to a public repository. For example, in addition to summary statistics, the data points behind means, medians and variance measures should be available. If there are restrictions on publicly sharing data—e.g. participant privacy or use of data from a third party—those must be specified.

Reviewer #1: Yes

**********

5. Is the manuscript presented in an intelligible fashion and written in standard English?

PLOS ONE does not copyedit accepted manuscripts, so the language in submitted articles must be clear, correct, and unambiguous. Any typographical or grammatical errors should be corrected at revision, so please note any specific errors here.

Reviewer #1: Yes

**********

6. Review Comments to the Author

Please use the space provided to explain your answers to the questions above. You may also include additional comments for the author, including concerns about dual publication, research ethics, or publication ethics. (Please upload your review as an attachment if it exceeds 20,000 characters)

Reviewer #1: Review comments to the Authors

I commend the authors for addressing my suggestions. However, there are still some minor issues that should be accounted for. I recommend a minor revision for now.

Comments:

I recommend that the p-value is reported with a lowercase italicized “p” throughout the text.

Lines 158-159: Since most individuals in the “wild” group were mostly rewilded rather than truly wild-hatched, the authors should provide a clearer justification for grouping them as “wild” as this may influence microbiome composition. Were all individuals collected in the private reserve previously reintroduced? Could they differ from truly wild individuals since hatching? I think a comparison between truly wild and headstarted individuals would be justified.

Line 232: Diversity is not the same thing as richness. The Shannon diversity index incorporates both species richness and evenness, but it is not a direct measure of richness alone. “richness” → “diversity”

Line 234: On Fig. 1A diversity is displayed, not richness Instead of “significantly richer,” I suggest “significantly more diverse.”

Lines 241-242: I think virus names should be italicized.

Lines 258-259: Fig.1, The phrasing of the title seems a little overcomplicated. I recommend revising the sentence to: “Diverse microbial taxa from multiple kingdoms identified in cloacal samples,” or something similar.

Lines 271-272: Be aware that these taxa are not all kingdoms, and are not at the same taxonomic level, so I recommend rewording the sentence

Line 282: In my opinion, it is better to write it as “p<0.001.”

Lines 330-331: I recommend recommend rephrasing the sentence, to avoid any overstatements. See Hernández et al., 2025.

Lines 338–339: “…that can influence overall organism health” — I think a few words about the link between microbial diversity and organismal health would strengthen the results.

Line 353: “phylum” instead of “phyla.”

Line 619: The references should be revised so that latin names are italicized.

Figure 1: Species names should be italicized.

Figure 2: There are dots in the legend, and it is not italicized. Also, the “p” should be lowercase.

References:

Hernández, M., Langa, J., Aizpurua, O., Navarro-Noya, Y. E., & Alberdi, A. (2025). Contrasting recovery of metagenome assembled genomes and derived bacterial communities and functional profiles from lizard fecal and cloacal samples. Animal Microbiome, 7(1), 15.

**********

7. PLOS authors have the option to publish the peer review history of their article (what does this mean?). If published, this will include your full peer review and any attached files.

If you choose “no”, your identity will remain anonymous but your review may still be made public.

**Do you want your identity to be public for this peer review?**  For information about this choice, including consent withdrawal, please see our Privacy Policy.

Reviewer #1: No

**********

[NOTE: If reviewer comments were submitted as an attachment file, they will be attached to this email and accessible via the submission site. Please log into your account, locate the manuscript record, and check for the action link "View Attachments". If this link does not appear, there are no attachment files.]

To ensure your figures meet our technical requirements, please review our figure guidelines: https://journals.plos.org/plosone/s/figures

You may also use PLOS’s free figure tool, NAAS, to help you prepare publication quality figures: https://journals.plos.org/plosone/s/figures#loc-tools-for-figure-preparation.

NAAS will assess whether your figures meet our technical requirements by comparing each figure against our figure specifications.

# Author comment: Author response to Decision Letter 2

**DOI:** 10.1371/journal.pone.0350824.r004

18 May 2026

Reviewer 1

I commend the authors for addressing my suggestions. However, there are still some minor issues that should be accounted for. I recommend a minor revision for now.

Our answer:

We thank the reviewer for their constructive feedback. We have revised the manuscript accordingly and made the appropriate changes.

Comment 1: I recommend that the p-value is reported with a lowercase italicized “p” throughout the text.

Response 1: Thank you for pointing this out. All p-values were changed and italicized in both the text and figures (please see updated manuscript and track changes document).

Comment 2: Lines 158-159: Since most individuals in the “wild” group were mostly rewilded rather than truly wild-hatched, the authors should provide a clearer justification for grouping them as “wild” as this may influence microbiome composition. Were all individuals collected in the private reserve previously reintroduced? Could they differ from truly wild individuals since hatching? I think a comparison between truly wild and headstarted individuals would be justified.

Response 2: Great point. All individuals in our study sampled at the private reserve were headstarted. We have run another analysis indicating there was no significant difference in community composition between headstarted individuals and true wild individuals (lines 218-220 in track changes document). Please also see attached supplemental figure S1 comparing headstarted individuals to truly wild individuals (lines 519-525 in track changes document). Furthermore, extrapolating from two previous lizard studies (Eliades et al. 2021 & Forehand et. al. 2025), cloacal microbiomes were indistinguishable between headstarted individuals and true wild individuals after 2 months post-release (References 11 and 19) which supports our findings too.

Comment 3: Diversity is not the same thing as richness. The Shannon diversity index incorporates both species richness and evenness, but it is not a direct measure of richness alone. “richness” → “diversity”

Response 3: Thank you for clarifying this. Changes were made (see lines 195, 222-225 & 376-377, 537) in track changes document).

Comment 4: Line 234: On Fig. 1A diversity is displayed, not richness Instead of “significantly richer,” I suggest “significantly more diverse.”

Response 4: Changed to, “wild snakes exhibited significantly greater Shannon diversity” (see line 224 in track changes document).

Comment 5: Lines 241-242: I think virus names should be italicized.

Response 5: All virus names are now italicized (lines 231-232 in track changes document).

Comment 6: Lines 258-259: Fig.1, The phrasing of the title seems a little overcomplicated. I recommend revising the sentence to: “Diverse microbial taxa from multiple kingdoms identified in cloacal samples,” or something similar.

Response 6: Title changed to: “Fig 1. Diverse microbial taxa from multiple kingdoms are observed in the cloacal samples” (Lines 248-249).

Comment 7: Lines 271-272: Be aware that these taxa are not all kingdoms, and are not at the same taxonomic level, so I recommend rewording the sentence.

Response 7: We changed to “across five major viral and microbial taxonomic groups”. (Lines 255-256 in track changes document).

Comment 8: Line 282: In my opinion, it is better to write it as “p<0.001.”

Response 8: Changed to P<0.001 (Line 262).

Comment 9: Lines 330-331: I recommend rephrasing the sentence, to avoid any overstatements. See Hernández et al., 2025.

Response 9: Changed from “reptilian cloacal microbiome” to “ophidian cloacal microbiome” (line 310 in track changes document).

Comment 10: Lines 338–339: “…that can influence overall organism health” — I think a few words about the link between microbial diversity and organismal health would strengthen the results.

Response 10: We agree. I’ve added this, “We have demonstrated that shotgun metagenomic sequencing provides valuable insight into the cloacal microbiome. This approach may also aid in identifying microbial associations relevant to disease status and overall snake health, as shifts in microbial diversity and community composition may reflect dysbiosis and alterations in immune function.” (Lines 318-321 in track changes document).

Comment 11: Line 353: “phylum” instead of “phyla.”

Response 11: Corrected (line 343 in track changes document).

Comment 12: Line 619: The references should be revised so that Latin names are italicized.

Response 12: All references are now revised so that Latin names are italicized (lines 558-783 in track changes document).

Comment 13: Figure 1: Species names should be italicized.

Response 13: Species names are corrected and now italicized (see Figure 1).

Comment 14: Figure 2: There are dots in the legend, and it is not italicized. Also, the “p” should be lowercase.

Response 14: Corrected (see Figure 2).

Attachment Submitted filename: Response_to_Reviewers_auresp_2.docx

# Editor report: Decision Letter 2

**DOI:** 10.1371/journal.pone.0350824.r005

**Authors:** Edvard Mizsei, Edvard Mizsei, Edvard Mizsei

20 May 2026

Cloacal microbiome variation in wild and captive Eastern Indigo Snakes (Drymarchon couperi) with and without Cryptosporidium serpentis infection

PONE-D-26-07884R2

Dear Dr. Brown,

We’re pleased to inform you that your manuscript has been judged scientifically suitable for publication and will be formally accepted for publication once it meets all outstanding technical requirements.

Within one week, you’ll receive an e-mail detailing the required amendments. When these have been addressed, you’ll receive a formal acceptance letter and your manuscript will be scheduled for publication.

An invoice will be generated when your article is formally accepted. Please note, if your institution has a publishing partnership with PLOS and your article meets the relevant criteria, all or part of your publication costs will be covered. Please make sure your user information is up-to-date by logging into Editorial Manager at Editorial Manager® and clicking the ‘Update My Information' link at the top of the page. For questions related to billing, please contact billing support.

If your institution or institutions have a press office, please notify them about your upcoming paper to help maximize its impact. If they’ll be preparing press materials, please inform our press team as soon as possible -- no later than 48 hours after receiving the formal acceptance. Your manuscript will remain under strict press embargo until 2 pm Eastern Time on the date of publication. For more information, please contact onepress@plos.org.

Kind regards,

Edvard Mizsei

Academic Editor

PLOS One

Additional Editor Comments (optional):

Reviewers' comments:

# Editor report: Acceptance letter

**DOI:** 10.1371/journal.pone.0350824.r006

**Authors:** Edvard Mizsei, Edvard Mizsei, Edvard Mizsei

PONE-D-26-07884R2

PLOS One

Dear Dr. Brown,

I'm pleased to inform you that your manuscript has been deemed suitable for publication in PLOS One. Congratulations! Your manuscript is now being handed over to our production team.

At this stage, our production department will prepare your paper for publication. This includes ensuring the following:

* All references, tables, and figures are properly cited

* All relevant supporting information is included in the manuscript submission,

* There are no issues that prevent the paper from being properly typeset

You will receive further instructions from the production team, including instructions on how to review your proof when it is ready. Please keep in mind that we are working through a large volume of accepted articles, so please give us a few days to review your paper and let you know the next and final steps.

Lastly, if your institution or institutions have a press office, please let them know about your upcoming paper now to help maximize its impact. If they'll be preparing press materials, please inform our press team within the next 48 hours. Your manuscript will remain under strict press embargo until 2 pm Eastern Time on the date of publication. For more information, please contact onepress@plos.org.

You will receive an invoice from PLOS for your publication fee after your manuscript has reached the completed accept phase. If you receive an email requesting payment before acceptance or for any other service, this may be a phishing scheme. Learn how to identify phishing emails and protect your accounts at https://explore.plos.org/phishing.

If we can help with anything else, please email us at customercare@plos.org.

Thank you for submitting your work to PLOS ONE and supporting open access.

Kind regards,

PLOS ONE Editorial Office Staff

on behalf of

Dr. Edvard Mizsei

Academic Editor

PLOS One
